# A Pyrene-4,5,9,10-Tetraone-Based Covalent Organic Framework Delivers High Specific Capacity as a Li-Ion Positive Electrode

**DOI:** 10.1021/jacs.2c02196

**Published:** 2022-05-19

**Authors:** Hui Gao, Alex R. Neale, Qiang Zhu, Mounib Bahri, Xue Wang, Haofan Yang, Yongjie Xu, Rob Clowes, Nigel D. Browning, Marc A. Little, Laurence J. Hardwick, Andrew I. Cooper

**Affiliations:** † Materials Innovation Factory and Department of Chemistry, University of Liverpool, 51 Oxford Street, Liverpool L7 3NY, U.K.; ‡ Stephenson Institute for Renewable Energy, Department of Chemistry, University of Liverpool, Peach Street, Liverpool L69 7ZF, U.K.; § Leverhulme Research Centre for Functional Materials Design, University of Liverpool, 51 Oxford Street, Liverpool L7 3NY, U.K.; ∥ Albert Crewe Centre, University of Liverpool, Waterhouse Building, Block C, 1-3 Brownlow Street, Liverpool L69 3GL, U.K.

## Abstract

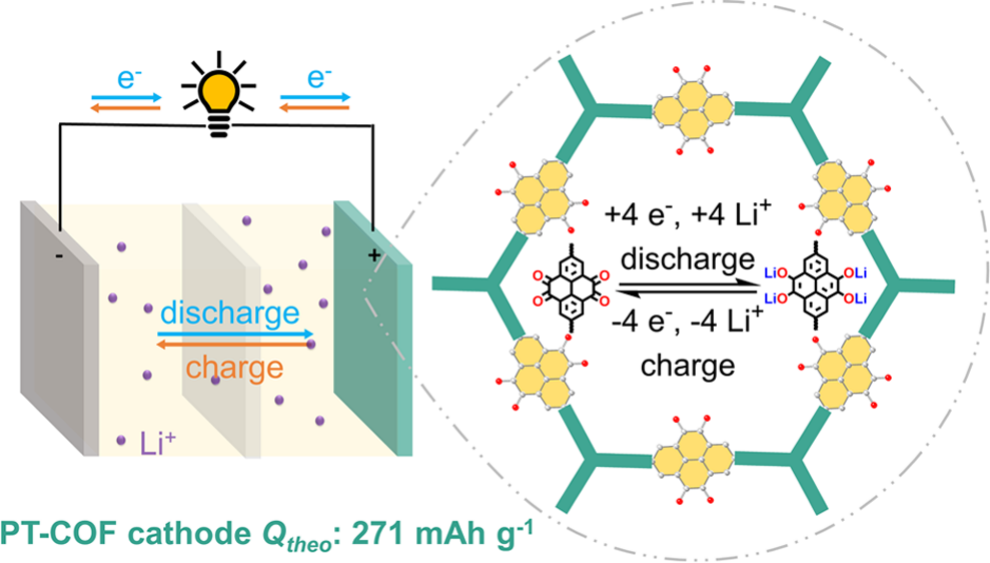

Electrochemically active covalent organic frameworks (COFs) are promising electrode materials for Li-ion batteries. However, improving the specific capacities of COF-based electrodes requires materials with increased conductivity and a higher concentration of redox-active groups. Here, we designed a series of pyrene-4,5,9,10-tetraone COF (PT-COF) and carbon nanotube (CNT) composites (denoted as PT-COFX, where *X* = 10, 30, and 50 wt % of CNT) to address these challenges. Among the composites, PT-COF50 achieved a capacity of up to 280 mAh g^–1^ as normalized to the active COF material at a current density of 200 mA g^–1^, which is the highest capacity reported for a COF-based composite cathode electrode to date. Furthermore, PT-COF50 exhibited excellent rate performance, delivering a capacity of 229 mAh g^–1^ at 5000 mA g^–1^ (18.5C). Using *operando* Raman microscopy the reversible transformation of the redox-active carbonyl groups of PT-COF was determined, which rationalizes an overall 4 e^–^/4 Li^+^ redox process per pyrene-4,5,9,10-tetraone unit, accounting for its superior performance as a Li-ion battery electrode.

## Introduction

Rechargeable Li-ion batteries are the mainstay of portable electronics and the rapidly growing electric vehicles sector,^1^ and improving their performance is hugely desirable. The theoretical capacities of electrode active materials restrict their performance in Li-ion batteries,^2^ and for organic electrodes to compete with conventional inorganic electrodes, new organic materials with higher specific capacities are needed. Additionally, electrode materials with good performance at higher rates and, therefore, with faster charging times are also needed. Unlike inorganic electrodes, organic materials have the advantage of being composed of lightweight elements and could be excellent candidates for lightweight applications, such as battery-powered aircraft.^3^ They also have tunable molecular structures and can, in some cases, be accessed from renewable sources.^4−6^ Another advantage is that simple redox reactions in organic electrode materials can occur on quick timescales, which could, in principle, lead to the discovery of batteries with better rate performances.^7,8^ Consequently, organic materials are promising candidate electrodes for the next generation of renewable Li-ion batteries with high capacity and rate performance. There are also intrinsic shortcomings that need to be overcome, however, such as poor intrinsic conductivity and undesirable solubility in electrolytes.^9^

Covalent organic frameworks (COFs) are crystalline materials that have modular structures and permanent porosity.^10^ One advantage of this modularity for battery applications is that redox-active units can be rationally incorporated to prepare COFs with improved electrochemical energy storage capacities.^11−16^ The well-defined and tunable permanent porosity in COFs can also enhance ion transport to active sites in their structures. COFs can also have better electrolyte stability than discrete organic molecules, which tend to be more soluble, leading to poor cycling stability.^17−19^ However, COFs often suffer from poor intrinsic conductivities and low utilization of their redox-active sites because a proportion of the active sites are deeply buried and inaccessible in the long (usually one-dimensional) channels.^17−21^ Hence, to improve the performance of COF-based electrodes, COF materials with higher theoretical capacities, enhanced conductivities, and optimized structures that permit facile access to the redox-active sites are needed.

**Figure 1:**
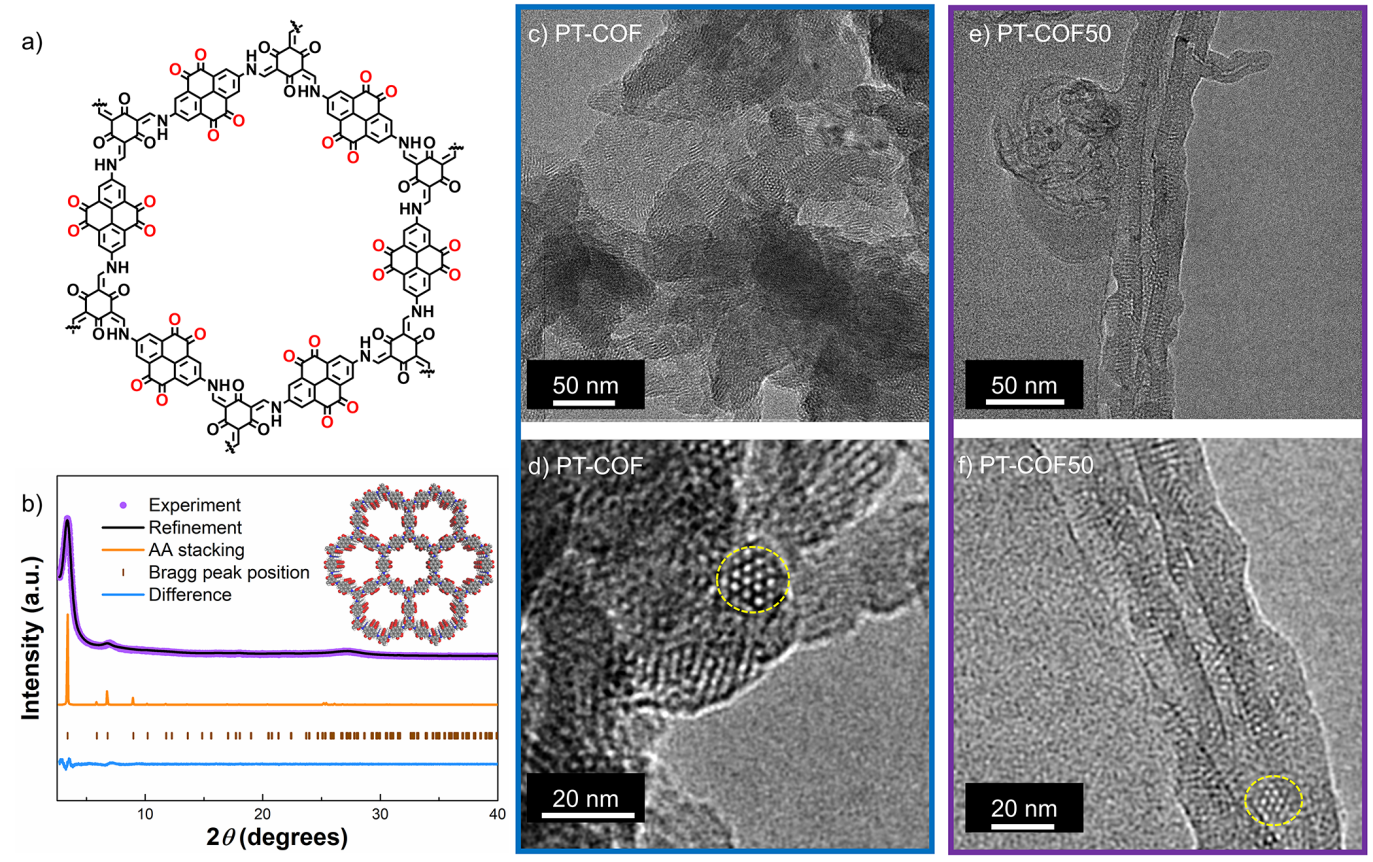
(a) Chemical structure of PT-COF. (b) PXRD pattern fitting of PT-COF with Pawley refinement (*a* = *b* = 30.07 Å, *c* = 3.55 Å, *V* = 2784.1 Å^3^, *R*_p_ = 2.21%, *R*_wp_ = 3.17%); inset shows AA stacked structural model for PT-COF along the crystallographic *c* axis (top). Atom colors: C, gray; N, blue; O, red; and H, white. (c,d) TEM images of PT-COF and (e,f) TEM images of PT-COF50. The circled regions highlight the comparable pore structures found in the TEM images of PT-COF and PT-COF50. Scale bars are included in the insets.

It was reported previously that only one carbonyl group per imide group in polyimides is redox-active in Li-ion batteries (Figure S1a in the Supporting Information, SI) because reducing the second carbonyl group requires potentials below 1.5 V vs Li^+^/Li, which leads in parallel to the decomposition of the structure.^22−25^ By contrast, all the carbonyl groups in phenanthraquinone and pyrene-4,5,9,10-tetraone derivatives can be utilized as redox-active sites in Li-ion positive electrodes without decomposing the structure (Figure S1b,c).^19^ For example, we recently reported a phenanthraquinone-containing COF that we used to form carbon nanotube (CNT) composite cathodes for Li-ion batteries, demonstrating 95% utilization of the carbonyl redox-active sites.^26^ However, although all the carbonyl groups were electrochemically redox-active and the COF/CNT composite allowed for ultrafast charging times, the overall capacity was limited by the 157 mAh g^–1^ theoretical specific capacity of the COF.^26^ Here, we designed composites containing a pyrene-4,5,9,10-tetraone COF (PT-COF, Figure 1a) that has a much higher concentration of redox-active carbonyl groups and a theoretical capacity that is ca. 73% higher (Figure S2); the same COF was also investigated recently as a supercapacitor.^27^ Composites of the PT-COF and CNT achieved specific capacities of up to 280 mAh g^–1^ as normalized to the active PT-COF material at 200 mA g^–1^. After subtracting capacity contributions from the CNT and carbon black components, this equates to up to 98% usage of the electrochemical redox-active sites of PT-COF. This capacity is the highest reported for a COF-based composite Li-ion positive electrode.^26^

## Results and Discussion

PT-COF was synthesized via a one-step Schiff-base condensation reaction of 2,7-diaminopyrene-4,5,9,10-tetraone (DAPT) and triformylphloroglucinol (TFG) in mesitylene and 1,4-dioxane (1:4 v/v) at 120 °C. The imine condensation reaction is followed by keto-enol tautomerization, which enhances the chemical stability of the COF.^28,29^ Full synthetic details and characterization data are provided in Supporting Information Section 3 and Figures S3–8. ^13^C CP-MAS solid-state NMR spectroscopy (Figure S9a) and FT-IR spectroscopy (Figure S9b,c) were used to confirm the formation of the keto-enol COF product. The PT-COF and CNT composites, PT-COF10, PT-COF30, and PT-COF50, were synthesized using the same method, but by adding 10, 30, and 50 wt % of CNT to reaction mixtures, respectively. As shown in Figure 1b, the powder X-ray diffraction (PXRD) pattern of PT-COF reveals that the COF is crystalline and that the pattern matches the simulated PXRD of the aligned AA stacked model. A Pawley refinement confirmed that the PXRD data were consistent with PT-COF having the same hexagonal *P*6/*m* symmetry and comparable dimensions to the eclipsed AA stacked model (Figure 1b). The addition of CNT into the COF reaction appeared to decrease the crystallinity of PT-COF in the PT-COFX composites somewhat, but the PXRD data indicated that PT-COF still retained the same AA stacked structure (Figure S10). PXRD and FT-IR also confirmed that the PT-COF exhibited good chemical stability in water, hydrochloric acid (1 and 12 M), *N*,*N*-dimethylformamide, and *N*-methyl-2-pyrrolidone after the PT-COF was immersed in each of these liquids for 48 h (Figure S11).

The surface area and porosity of PT-COF and the PT-COFX composites were measured by N_2_ adsorption–desorption analysis at 77.3 K. PT-COF, PT-COF10, PT-COF30, and PT-COF50 have Type II isotherms (Figure S12) with Brunauer–Emmett–Teller (BET) surface areas of 432, 450, 473, and 318 m^2^ g^–1^, respectively. The pore size distributions of PT-COF and the PT-COFX composites, derived by fitting nonlocal density functional theory (NL-DFT) models to the N_2_ isotherms, were ∼1.8 nm (Figure S12), which is close to the pore size in the AA stacked model (∼2.3 nm).

The morphologies of PT-COF and the PT-COFX composites were characterized by transmission electron microscopy (TEM). TEM further confirmed the crystalline structure of the PT-COF. The TEM images showed that PT-COF has an ordered structure with hexagonal-shaped pores (areas outlined in yellow in Figure 1c,d). The TEM images of PT-COF50 (Figure 1e,f), PT-COF10 (Figure S13a,b), and PT-COF30 (Figure S13c,d) show that the PT-COF retains its crystalline structure in the PT-COFX composites.

### Electrochemical Properties

The PT-COF and the PT-COFX composites were studied as positive electrodes in Li-ion coin cells using Li metal as the counter electrode. The electrolyte of 1 M lithium bis(trifluoromethanesulfonyl)imide (LiTFSI) in dioxolane (DOL) and dimethoxyethane (DME) (1:1 v/v) was used instead of the electrolyte of 1 M lithium hexafluorophosphate (LiPF_6_) in ethylene carbonate (EC) and dimethyl carbonate (DMC) (1:1 v/v). This was because there might be undesired side reactions of the active sites with the carbonate-based electrolyte (Figure S14).^30,31^

**Figure 2:**
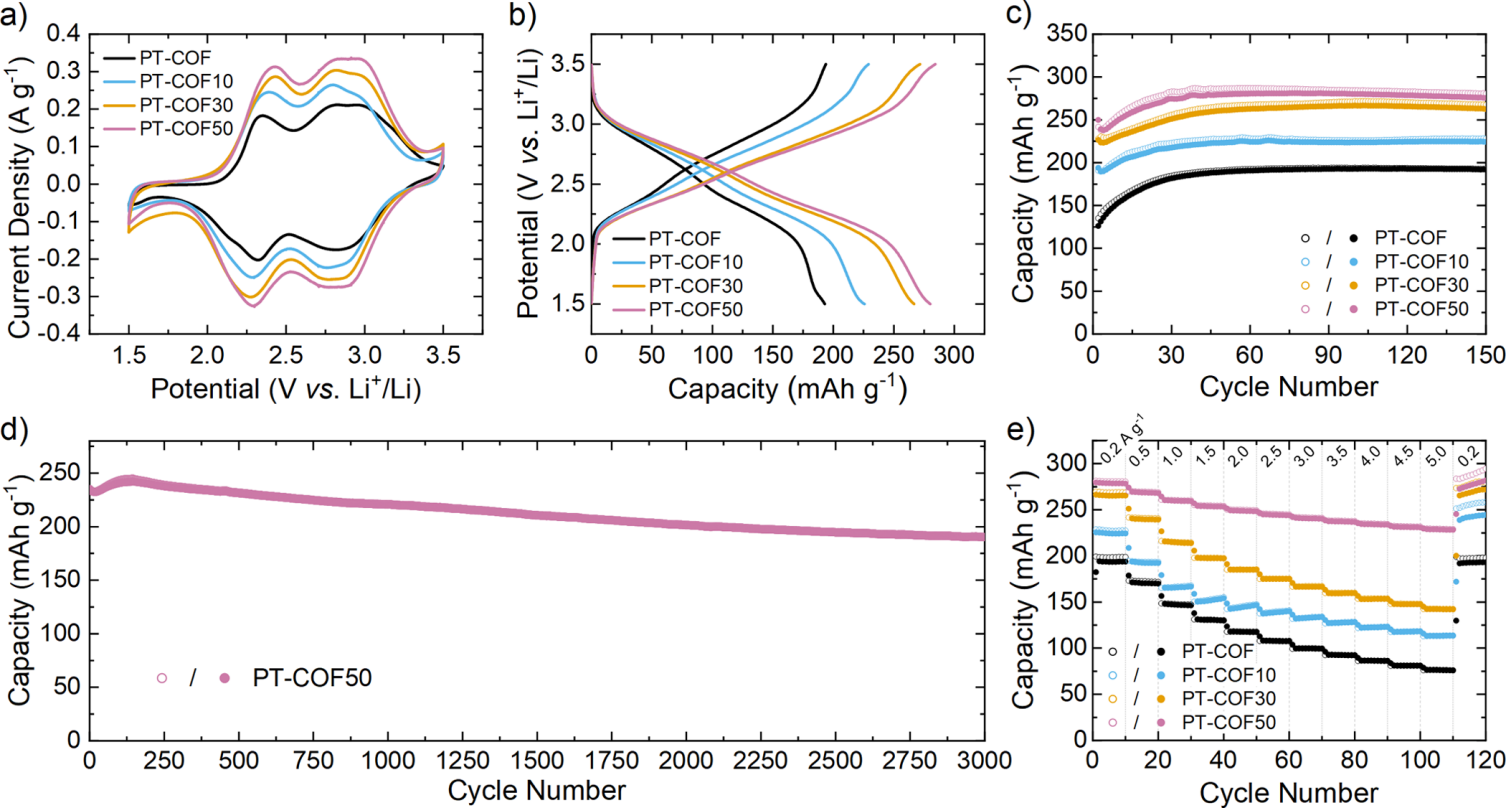
(a) CV profiles at a scan rate of 0.5 mV s^–1^; (b) charge–discharge profiles at 200 mA g^–1^; (c) cycling performances over 150 cycles at 200 mA g^–1^; (d) long cycling performance of PT-COF50 at 2000 mA g^–1^; (e) rate performance of PT-COF, PT-COF10, PT-COF30, and PT-COF50. Open symbols represent the charge capacity, and solid symbols represent the discharge capacity.

Cyclic voltammetry (CV) of PT-COF and the PT-COFX composites was performed in the coin cells at 0.5 mV s^–1^ in a potential window of 1.5–3.5 V (Figure 2a). All the curves have similar shapes in the CV profiles. Four separated reduction peaks were present at ca. 2.9, 2.7, 2.3, and 2.1 V, which correspond to the redox reaction of the four carbonyl groups in the DAPT unit (discussed in the following section). Furthermore, with an increasing amount of CNT in the composite, the peak current densities and integral charge (based on the mass of PT-COF active material) increased. PT-COF50 has the highest integral charge area, implying greater utilization of redox-active sites in PT-COF50 than in PT-COF. This may be because the charge transport within the composite electrode was improved by adding CNTs.

Galvanostatic charge–discharge tests were performed in a 1.5 to 3.5 V voltage window for all the Li-ion coin cells (Figure 2b). The sloping plateaus in the galvanostatic charge/discharge curves are consistent with the CV profiles of the electrodes, corresponding to the reversible oxidization/reduction of the carbonyl groups. The average discharge potential calculated from the PT-COF and the PT-COFX composites is around 2.55 V, which is higher than most other carbonyl functionalized organic electrodes.^16,31−35^ PT-COF delivered a specific capacity of 193 mAh g^–1^ at 200 mA g^–1^, corresponding to 71% of its theoretical capacity of 271 mAh g^–1^ (see Supporting Information Section 2 for full details). By contrast, the specific capacities of the PT-COFX composites increased up to a maximum of 280 mAh g^–1^ in PT-COF50 (Table 1). After subtracting capacity contributions from the CNT and carbon black components, this capacity equates to a 98% utilization of redox-active sites in the PT-COF50 electrode. Therein, the capacity contributions of CNTs in the composites were only 1, 6, and 13 mAh g^–1^ for PT-COF10, PT-COF30, and PT-COF50, respectively (Table S3). Moreover, after a steady increase over the first ca. 30 cycles, the PT-COF and the PT-COFX composite electrodes retained a near-constant capacity after 150 cycles (Figure 2c). The initial increase in deliverable capacity in the first ca. 30 cycles may be attributed in part to the complete wetting of internal COF pores, thus improving access to redox-active sites. The PT-COF50 composite additionally exhibited excellent long-term cycling stability, retaining 82% of its initial capacity after 3000 cycles at 2000 mA g^–1^ (Figure 2d). Notably, PT-COF and PT-COFX composites had far superior cycling performance than the DAPT monomer-based electrode (Figure S15). As shown in Table S4, the capacity of PT-COF50 outperforms that of other related COF-based composite cathodes.^12−14,16,17,26,32,33,36,37^

**Table 1:** Electrochemical Performance of PT-COF, PT-COF10, PT-COF30, and PT-COF50

electrode	*Q*^a^/mAh g^–1^	active site utilization^b^ (%)	rate performance	
			*Q*_maxJ_^c^/mAh g^–1^	retention^d^ (%)
PT-COF	193	71	76	39
PT-COF10	225	83	115	51
PT-COF30	267	96	145	54
PT-COF50	280	98	229	82

a The highest delivered reversible discharge capacity (*Q*) at 200 mA g^–1^.

b Subtracted the capacity contribution of CNTs.

c Capacity at a current density of 5000 mA g^–1^ (*Q*_maxJ_).

d Capacity retention at 5000 mA g^–1^ related to 200 mA g^–1^.

The electrochemical performance of pure CNT and carbon black was also measured to determine their capacity contributions toward the overall performance of the electrodes. The electrodes of the CNT and carbon black exhibit capacities of 13 and 2 mAh g^–1^ under 200 mA g^–1^, when the electrode is made from CNT:PVDF (9:1 by mass) and carbon black:PVDF (9:1 by mass), respectively (Figure S16).

The overall capacities of PT-COF, PT-COF10, PT-COF30, and PT-COF50 cells are 128, 158, 145, and 109 mAh g^–1^, respectively, when calculated based on the combined mass of the active material and the conductive additives (Figure S17). PT-COF50 shows the lowest capacity when considering the mass of the whole electrode. This demonstrates that adding too much conductive additives is a poor strategy for practical cells. Here, we focus on the material-specific electrochemical properties of PT-COF, and, consequently, all the gravimetric capacities and currents are normalized to the mass of the active material (PT-COF).

The rate performance of PT-COF and the PT-COFX composites was then studied under different current densities over the range of 200 to 5000 mA g^–1^ (Figure 2e and Table 1). The capacity of all composites recovered to the initial values once the current was reduced back to 200 mA g^–1^. The capacity of PT-COF50 is the least dependent on the current density, achieving a capacity retention of 82%, corresponding to the capacity of 229 mAh g^–1^, at 5000 mA g^–1^ (equating to 18.5C, where the 1C = 271 mA g^–1^ derived from the theoretical capacity of the PT-COF). Therein, this capacity equates to a utilization of the carbonyl redox-active sites of 78%, even at the high current density of 5000 mA g^–1^. These results indicate good rate performance. Simulation of the electrochemical impedance spectra of the PT-COF and the PT-COFX composite-based cells (Figure S18) was used to extract the impedance characteristics (Table S5) of the different composites before electrochemical cycling. Critically, the calculated charge transfer resistance of the PT-COF50 cell (27.2 Ω) was found to be significantly reduced compared with PT-COF (189 Ω) and the PT-COF10 and PT-COF30 composites (164 and 100 Ω, respectively). This results in the dramatically enhanced rate performance of PT-COF50 compared to PT-COF and the PT-COF10 and PT-COF30 composites. The rate capability of PT-COF50 outperforms that of some recently reported COF cathodes. For example, a two-dimensional (2D) boroxine-linked chemically active pyrene-4,5,9,10-terarone COF (PPTODB), which contains the same electrochemical redox motif with PT-COF, had a capacity retention of less than 50% at 1500 mA g^–1^.^37^ PIBN-G delivered a capacity of 271 mAh g^–1^ at 0.1C and showed 73% of this capacity at 10C.^33^

### Mechanistic Investigations

To probe the mechanism underlying charge storage in the PT-COF electrodes, ex situ Fourier-transform infrared (FT-IR) spectroscopy was first used to characterize bulk material changes before and after cycling. In the FT-IR spectra presented in Figure S19, the peak at 1675 cm^–1^ characteristic of the C=O groups in the DAPT structure disappeared when fully discharged to 1.5 V vs Li^+^/Li. Encouragingly, the C=O bond feature reforms when the electrode is recharged, and the spectrum of the charge electrode appears almost identical to that of the pristine electrode. This observation, consistent with our earlier work on the analogous 2-carbonyl 9,10-phenanthrequinone-based COF,^26^ supports that the reversible charge storage involves the redox electrochemistry of the carbonyl (C=O) of the DAPT structure. For the 4-carbonyl containing PT-COFX composite materials, the earlier CVs (Figure 2a) indicate two prominent reaction peaks separated by ca. 0.5 V, wherein both of these primary peaks are further constituted by two peaks close in energy (most distinctly for the first redox couple observed on the negative sweep at ca. 2.9 V). This indicates a four-step process (corresponding with the redox reactions involving the four carbonyl groups on the DAPT unit). Conversely, CVs of the DAPT monomer (Figure S20) reveal only two distinct, and much sharper, peaks within the voltammograms. Furthermore, the thermodynamic redox potentials for the free monomer are also lower than those measured for the COF. These observations suggest that the structural properties of the rigid COF structure may alter the reaction pathways, and associated energy levels, during (de)lithiation. Kinetic analyses of the carbonyl redox reactions in the PT-COF and PT-COF50 electrodes, utilizing CV peak currents (0.1–1 mV s^–1^, see Figure S21 in the Supporting Information for full description), indicate that the redox chemistry for both materials appears as highly surface-controlled, not limited by electrolyte diffusion within the studied range. Therein, the b values for PT-COF50 were slightly larger (closer to one), suggesting a structure more optimized for faster, surface-controlled redox reactions.

**Figure 3:**
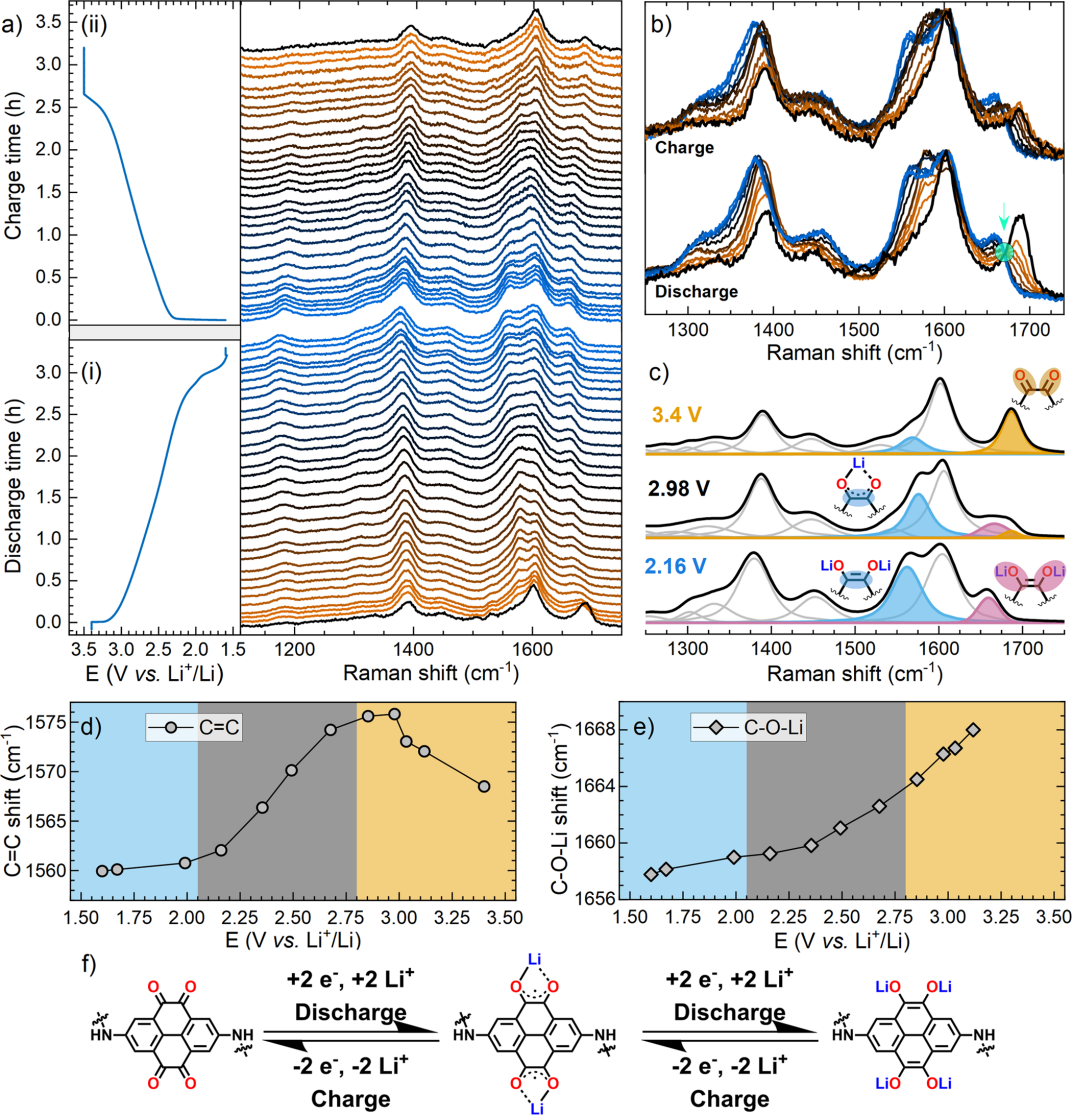
*Operando* Raman microscopy of a free-standing PT-COF electrode during galvanostatic discharging (a-i, lithiation) and charging (a-ii, delithiation) between 3.5 and 1.5 V vs Li^+^/Li at 54 mA g^–1^ (C/5). (b) Selected spectra from discharge and charge steps showing the primary shifts/growths in peaks of interest and the observed isosbestic point highlighted by the turquoise circle (ca. 1670 cm^–1^). (c) Example fitted spectra used to extract peak characteristics for the changes observed in (d) aromatic C=C bond and (e) the lithium enolate C–O–Li mode as a function of discharge. (f) Proposed 4 e^–^ (4 Li^+^) reversible electrochemical redox mechanism of PT-COF during the lithiation/delithiation process.

To further characterize the reaction process, *operando* Raman microscopy was used to track structural changes in the PT-COF material during discharge and charge.^38,39^ The study, summarized in Figure 3, used a free-standing porous PT-COF electrode cycled galvanostatically versus a Li foil counter electrode in parallel with spectral acquisition, well-representing the true conditions of electrochemical cells discussed earlier. Selected spectra presented stacked as a function of the depth of the discharge (Figure 3a-i), following baseline subtraction and normalization, reveal the evolution of new and shifting bands during discharge that are reversed upon charging of the electrode (Figure 3a-ii). The primary changes in the measured spectra relate to changes in the C=O stretching mode (1686 cm^–1^) and the band growth/shift at 1576–1560 cm^–1^, relating to the formation of a C=C mode of varying delocalization and aromatic character. A slight downward shift is also observed in the peak at 1390 cm^–1^, attributed to the increasing aromaticity of the C=C bonds within the pyrene structure as the discharge proceeds. Selected spectra were fitted to extract information on peak splitting and shifting as a function of the discharge process (Figure 3c, see Figure S22 for all fitted spectra). Therein, the assigned C=O stretching mode reduces in intensity on discharge, in agreement with a report on in situ FT-IR spectroscopy of a related non-COF polymer material.^40^ However, we also observed that this C=O mode splits with the formation of a lower wavenumber band (1658 cm^–1^) assigned to the developing lithium enolate character (C–O–Li, Figure 3f). In line with the complete loss of the pure C=O mode below, ca. 2.8 V, a well-defined and consistent isosbestic point (1670 cm^–1^, Figure 3b) is observed between these two modes, indicating the relation between the two species as the discharge reaction proceeds. The lithium enolate peak intensity grows as a function of the discharge, progressively shifting to lower wavenumbers until close to the end of the main discharge voltage plateau at ca. 2–2.2 V (Figure 3e).

The assigned C=C mode (1576–1560 cm^–1^) intensity increases during discharge until ca. 2.5 V. In parallel, the fitted peak location undergoes a transition as a function of the discharge state, first shifting to higher wavenumbers from 1568 cm^–1^ at 3.4 V to 1576 cm^–1^ at 2.8 V (Figure 3d), corresponding with the complete loss of the C=O stretching mode below 2.8 V during discharge (described above). The C=C peak then shifts to progressively lower wavenumbers as the discharge proceeds, leveling out to 1560 cm^–1^ toward the end of the primary plateau at ca. 2–2.2 V, where the bulk lithiation process is complete. This transition in peak shifts can be ascribed to the transition between the two main reaction steps highlighted in Figure 3f. Initially, moving from the 1,2-diketone group (2 groups per pyrene-4,5,9,10-tetraone unit) to the 1 e^–^ reduced form, consisting of delocalized charge sharing coordination of 1 Li^+^ cation per group. Subsequently, confirmed by the loss of pure C=O signals at ca. 2.8 V and the concurrent downward shift of the C=C bond character below this voltage, the second e^–^ reduction (per group) generates the final di-lithium enolate groups (2 per PT unit). The downward shift in the C=C bond as the discharge reaction proceeds from 2.8 to ca. 2 V arises from the overall aromaticity of the pyrene ring network formed in the final reduced product.

While the Raman investigations reveal mechanistic and product information for the two main 2 e^–^ redox processes for PT-COF electrodes, the observed peak splitting in the CVs of the PT-COFX composite electrodes (discussed earlier, Figure 2a) implies that the redox process observed during cycling would be better described as a four-step process. Critically, this is different from the constituent monomer, which, from the sharp and well-defined CV peaks, clearly undergoes two 2 e^–^ reductions. We, therefore, postulate that the crystalline organization and rigidity of the PT-COF, especially within the plane of an individual layer, causes a splitting of each 2 e^–^ step. This most notably increases the overpotential of introducing the second Li^+^ cation to the PT unit (given 2 distinct CV peaks are herein clearly identifiable). First, we consider the PT-COF layers simply as a continuous series of tessellating hexagons containing 1 PT unit per edge (i.e., 6 PT units per hexagon resulting in 6 inner and 6 outer 1,2-diketone groups, illustrated in Figure S23). For the first electron reduction, to maximize charge separation in the hexagonal structures, three nonadjacent PT units (i.e., edges 1, 3, and 5 of hexagon A) accept 1 Li^+^ cation inside the ring and the remaining 3 (i.e., edges 2, 4, and 6) accept 1 Li^+^ cation outside the ring (repeating across the COF plane). For the second e^–^ reduction, where we observe a small energy barrier in the CVs, the second Li^+^ cation per PT unit is introduced to the opposite side of the PT unit. Therefore, the Li^+^ cation must coordinate, during the reduction, to a 1,2-diketone unit (e.g., edge 2) that is adjacent to 2 already reduced groups (e.g., edges 1 and 3). This spatial distribution of charge from the reduced groups surrounding the actively reacting groups at each step could contribute to the observed energy barrier. This Li^+^ coordination to reducing species must also involve some degree of desolvation, given the COF pore/channel size of 1.8 nm accepts 3 Li^+^ cations per step (12 cations per internal hexagonal COF unit channel), and the size of solvation species [Li(*DME*)_*X*_]^+^ is in the region of 0.7–0.3 nm for *X* = 3 and 1, respectively.^41^ Such contributions could only arise from the rigid superstructure of the PT units in the COF and, thus, would not be expected from the discreet, redox-active DAPT monomer.

In addition to the differences between the voltammetry of COF and monomeric forms of the active material (discussed earlier), we also observed an interesting phenomenon within the Raman spectra of the PT-COF with and without the electrolyte. The C=O stretching mode of the (1686 cm^–1^) in the dry, pristine PT-COF shifted negatively by ca. 10–12 cm^–1^ when measured wetted with the electrolyte (see Figure S24). Conversely, this shift between dry and wetted is not observed at all for the DAPT monomer, again suggesting that the rigid organization of the active unit into the COF structure greatly affects interactions with the electrolyte. This shift in the C=O mode observed in the dry/wetted PT-COF is comparable to that observed during the discharge process in the *operando* electrochemical cell. Critically, however, the shift is not coupled with the observed changes relating to the C=C modes at 1576–1560 cm^–1^ that arise from the electrochemical reduction process. Therein, it is suggested that the shift in the Raman mode arises from weak coordination with the partially desolvated Li^+^ cations as the electrolyte diffuses into the COF pores. These shifts (also observed in the wetted electrode at open-circuit potential in the *operando* cell) are reversed by positive polarization of the working electrode, as can be seen by the first spectra collected at 3.4 V (*t* = 0 h in the discharge, highlighted in Figure S24).

## Conclusions

A COF-based electrode with a high specific capacity was designed by tuning the molecular structure of the COF and forming composites with CNTs. The utilization of the redox-active sites in the PT-COF-based electrode, which was 71% in the pure COF electrode, increased to 98% in the PT-COF50 composite containing 50 wt % of CNT. This utilization of active sites enabled PT-COF50 to deliver an ultrahigh capacity of 280 mAh g^–1^ at 200 mA g^–1^ as normalized to the active material, which is the highest value so far among COF-based composite electrode materials to date, to the best of our knowledge. The optimized composite electrode also displayed excellent rate performance, retaining 82% capacity (translating to 229 mAh g^–1^) at high currents of 5000 mA g^–1^. The remarkable performance of PT-COF50 was made possible by the inclusion of four redox-active sites per unit in the PT-COF, with the addition of CNT shown to improve the accessibility of active sites significantly.

To rationalize the performance of the PT-COF50 composite, we utilized *operando* Raman microscopy, which revealed the primary structural changes and key transition steps for electrochemical reactions of the carbonyl groups of the COF electrode in the Li^+^-containing electrolyte. In addition, voltammetry of the PT-COF materials indicates a four-step (4 e^–^/Li^+^) process, and a mechanism was proposed taking into consideration the effects of the rigid superstructure of the crystalline COF.

Organic electrode materials have the potential to achieve high capacity by designing the chemical structure. However, the present drawback of organic electrodes is that a large amount of conductive additive is required. Improving the specific capacity of organic electrode materials would accelerate organic electrode materials toward practical use, for example, by designing porous fully conjugated COFs with higher intrinsic conductivities, which would reduce the need for conductive additives.

## Supporting Information Available

Experimental details, synthetic methods, and characterization data (FT-IR, PXRD, TEM, solid-state NMR, N_2_ gas sorption isotherms and pore size distributions, and Raman microscopy) for PT-COF and the PT-COFX composites. Atomic coordinates and unit cell parameters of the AA model for PT-COF. Cycling performance of PT-COF in a conventional Li-ion battery electrolyte (1 M LiPF_6_ in EC/DMC (1:1 v/v)). Cycling performance of DAPT monomer. Electrochemical performance of CNT and carbon black. Charge–discharge profiles of PT-COF and the PT-COFX composites were calculated based on the sum mass of active materials, CNT, and carbon black. Electrochemical impedance spectroscopy of the PT-COF and PT-COFX electrodes. Ex situ FT-IR characterization of PT-COF electrode before and after electrochemical cycling. CV of the DAPT monomer. Kinetic analysis of the CVs of PT-COF and PT-COF50. Fitted *operando* Raman spectra of the PT-COF working electrode. Proposed rationalization of the 4 step discharge mechanism of PT-COF. Raman spectra of PT-COF and DAPT-monomer electrodes with and without wetting by the electrolyte.(PDF)
