# Supplementary material for: A Pyrene-4,5,9,10-Tetraone-Based Covalent Organic Framework Delivers High Specific Capacity as a Li-Ion Positive Electrode
Source: J Am Chem Soc. 2022 May 19;144(21):9434–42. doi: 10.1021/jacs.2c02196 (PMC9164232; doi:10.1021/jacs.2c02196)
Supplement: Supplementary file 1 — ja2c02196_si_001.pdf [file ja2c02196_si_001.pdf]

A Pyrene-4,5,9,10-tetraone-based Covalent Organic Framework Delivers High Specific Capacity as a Li-ion Positive Electrode

Hui Gao,[a,b,†] Alex R. Neale,*[b] Qiang Zhu,[a,c] Mounib Bahri,[d] Xue Wang,[a,c] Haofan Yang,[a,c] Yongjie Xu,[a,c] Rob Clowes,[a] Nigel D. Browning,[d] Marc A. Little,*[a] Laurence J. Hardwick*[b] and Andrew I. Cooper*[a,c]

[a] Materials Innovation Factory and Department of Chemistry, University of Liverpool, 51 Oxford Street, Liverpool L7 3NY, UK.

[b] Stephenson Institute for Renewable Energy, Department of Chemistry, University of Liverpool, Peach Street, Liverpool L69 7ZF, UK.

[c] Leverhulme Research Centre for Functional Materials Design, University of Liverpool, 51 Oxford Street, Liverpool L7 3NY, UK.

[d] Albert Crewe Centre, University of Liverpool, Waterhouse Building, Block C, 1-3 Brownlow Street, L69 3GL, UK.

Contents

1. Materials and Methods 2

2. Theoretical Specific Capacity Calculation 4

3. Synthetic Procedures 6

4. Supplementary Data 15

5. Reference 36

1. Materials and Methods

1.1 Materials

Dichlorodicyanobenzoquinone (DDQ) and 1,3,5-triformylphloroglucinol (TFG) were obtained from Tokyo Chemical Industry UK Ltd. Pyrene, sodium periodate, ruthenium (III) chloride hydrate, sodium dithionite, ammonium chloride, acetic acid, multi-walled carbon nanotubes with outer diameter and length ranges of 7–15 nm and 0.5-10 µm (CNTs), anhydrous *N*-methyl-2-pyrrolidone (NMP), dibutyl phthalate, and polyvinylidene fluoride (PVDF) were purchased from Sigma Aldrich. 1,4-dioxane was obtained from Alfa-Aesar. Fuming nitric acid and mesitylene were obtained from Acros Organics. Concentrated sulfuric acid, dimethylformamide (DMF), dimethyl sulfoxide (DMSO), dichloromethane (DCE), acetonitrile, chloroform, acetone, sodium hydroxide, sodium hydrogen carbonate, ethyl acetate, and methanol were obtained from Fisher. All chemicals were used as received without further purification.

1.2 Methods

Solution Nuclear Magnetic Resonance (NMR): ^{1}H and ^{13}C NMR spectra were recorded on a Bruker Avance 400 NMR spectrometer at 400 MHz and 100 MHz, respectively. The NMRs were referenced against the ^{1}H or ^{13}C signal of the solvent.

Solid-state NMR: ^{13}C magic-angle spinning measurements were carried out at 100 MHz using a Bruker Avance III HD spectrometer and a 4 mm (rotor o.d.) probe. Spectra were acquired at a spin rate of 10 kHz. Cross-polarisation spectra were recorded with total suppression of spinning sidebands, 1 ms contact time and with a recycle delay of 4 s. Carbon spectral referencing is relative to neat tetramethylsilane, carried out by setting the high-frequency signal from an external sample of adamantane to 38.5 ppm.

Powder X-ray Diffraction (PXRD): PXRD patterns were collected at room temperature in vertical transmission mode from loose powder samples held on Mylar film in aluminum well plates, using a Panalytical Empyrean diffractometer equipped with a high throughput screening XYZ stage, X-ray focusing mirror, and PIXcel detector with Cu-Kα (λ = 1.541 Å) radiation.

Fourier-transform Infrared Spectroscopy (FT-IR): FT-IR spectra of the as-prepared samples were recorded on a Bruker alpha spectrometer by attenuated total reflectance.

FT-IR spectra of the PT-COF electrode were recorded after discharging and charging the electrode to determine lithiation sites. The lithiated and delithiated electrodes were recovered from the cycled cells. The recovered electrodes were washed by DOL and dried under reduced pressure at room temperature, and FT-IR spectra were then recorded inside the glovebox

Gas Sorption Analysis: Surface area and N_2 sorption isotherms were measured at 77.3 K using a Micromeritics ASAP 2020 volumetric adsorption analyzer. Before analysis, samples were degassed at 120 °C for 12 hours under vacuum (10^{-5} bar). Surface areas were calculated over the relative pressure range (P/P_0) 0.05 to 0.12 using the Brunauer-Emmet-Teller (BET) surface area method. Pore size distributions were modelled by fitting the nonlocal density functional theory (NL-DFT) to the adsorption data.

Transmission Electron Microscopy: TEM images were obtained using a JEOL 2100+ microscope operating at 200KV equipped with a Gatan Rio Camera. The samples were prepared by drop-casting sonicated ethanol suspensions of the materials onto copper grids.

Raman Microscopy: Raman spectra were collected using a Renishaw In-Via Raman spectrometer equipped with an inverted microscope and a 50x objective. The excitation wavelength was a 532 nm wavelength laser and the samples were measured at room temperature. Galvanostatic cycling of the *operando* spectroelectrochemical cell was controlled using a Biologic SP-150 potentiostat (see Supporting Information Section 3.9 for full details)

Cyclic Voltammetry (CV): CV was recorded starting from open circuit potential to the cathodic scan (discharge) direction using a Biologic MPG2 potentiostat.

Electrochemical Impedance Spectroscopy (EIS): EIS was carried out on Biologic over a frequency range from 1 MHz to 100 mHz, with an applied sinusoidal voltage amplitude of $\pm$10 mV.

Galvanostatic Discharge-charge: cycling of the cells was measured using a Maccor Series 4000 battery cycler. All potentials are given versus Li^+/Li. All electrochemical measurements were performed on the coin cells at 25 °C. The cells were rested at 25 °C for 3 h before commencing the galvanostatic charge/discharge measurements.

2. Theoretical Specific Capacity Calculation

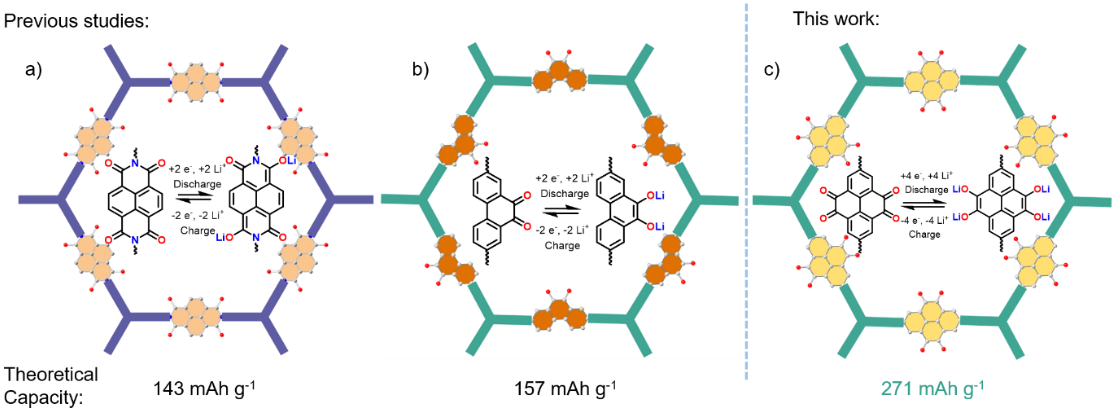

Figure S1. The theoretical capacity of different COFs.

$$C_{\mathrm{spec}} = \frac{n\times \mathrm{F}}{M_{\mathrm{w}}} = \frac{n\times 26.8\ (\mathrm{Ah\ mol^{-1}})}{M_{\mathrm{w}}\ (\mathrm{kg\ mol^{-1}})} \qquad \textbf{Equation S1}$$

Where n is the number of electrons transferred per redox reaction. F represents the Faraday constant, and M_w is the molar weight of the repeat unit of the organic component.

The repeating unit in PT-COF consists of 1/2 of a A unit (M_A) and 1/3 of a B unit (M_B) (Figure S2). Hence, the molecular weight of the repeating unit cell in PT-COF is 198.02 g mol^{-1} ($C_{11}H_4NO_3$). The number of electrons (n) involved in the repeating unit is equal to 2, assuming 4 electrons are involved in each phenanthrenequinone unit. Therefore, using Equation S1, the theoretical capacity of PT-COF is calculated to be 271 mAh g^{-1}.

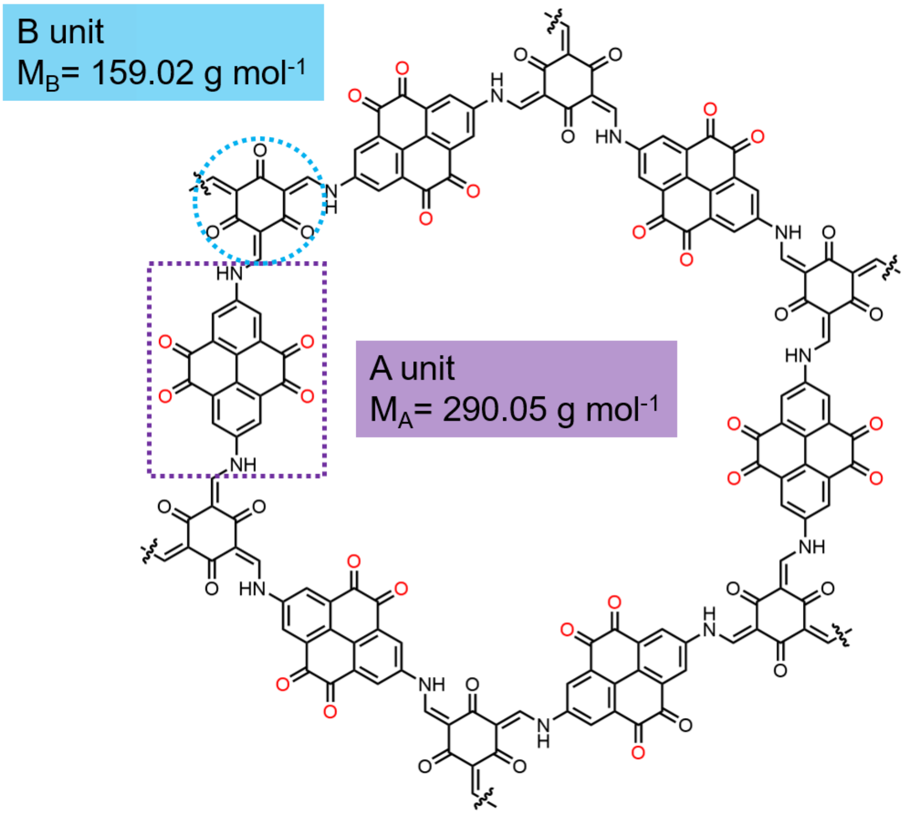

Figure S2. Chemical structure of PT-COF.

3. Synthetic Procedures

3.1 Synthesis of Pyrene-4,5,9,10-tetraone (PT)

PT was synthesized using a previously reported method.[1,2] In a 500 mL round flask equipped with a condenser and a stirrer bar, pyrene (20 mM, 4.0 g) was dissolved in a mixture of 80 mL dichloromethane and 80 mL acetonitrile. Then, sodium periodate (164 mM, 35.0 g), 100 mL H_2O, and Ruthenium (III) chloride hydrate (0.42 mM, 0.5 g) were added to the mixture. The reaction mixture was stirred at 35-40 °C overnight. After the reaction, the suspension was removed by filtration, and the precipitate was washed with copious amounts of DCM. The filtrate was extracted by DCM several times. A yellow solid was obtained by rotary evaporation of the organic solution. Then, the crude product was recrystallized from chloroform to afford 1.2 g of PT: 22.9% yield. ^1H NMR (400 MHz, DMSO-d_6, δ ppm): 8.33 (d, J_{HH} = 7.5 Hz, 4H), 7.75 (t, J_{HH} = 7.5 Hz, 2H). ^{13}C NMR (100 MHz, DMSO-d_6, δ ppm): 177.5, 134.6, 134.0, 131.8, 130.7. HRMS (EIS): calcd. for $([C_{16}H_6O_4]+Na)^+$: m/z = 285.206; found: 285.0162.

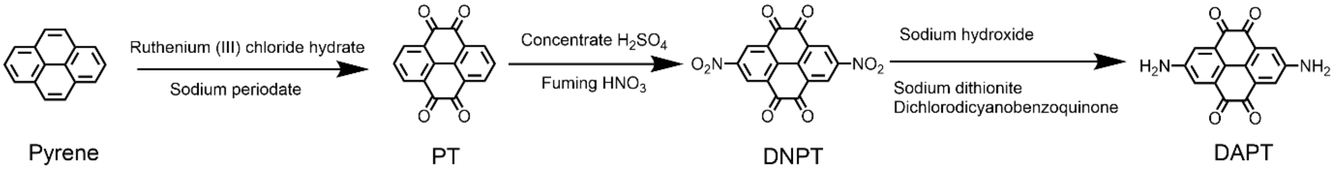

Scheme S1. Synthetic routes of small molecules PT, DNPT and DAPT.

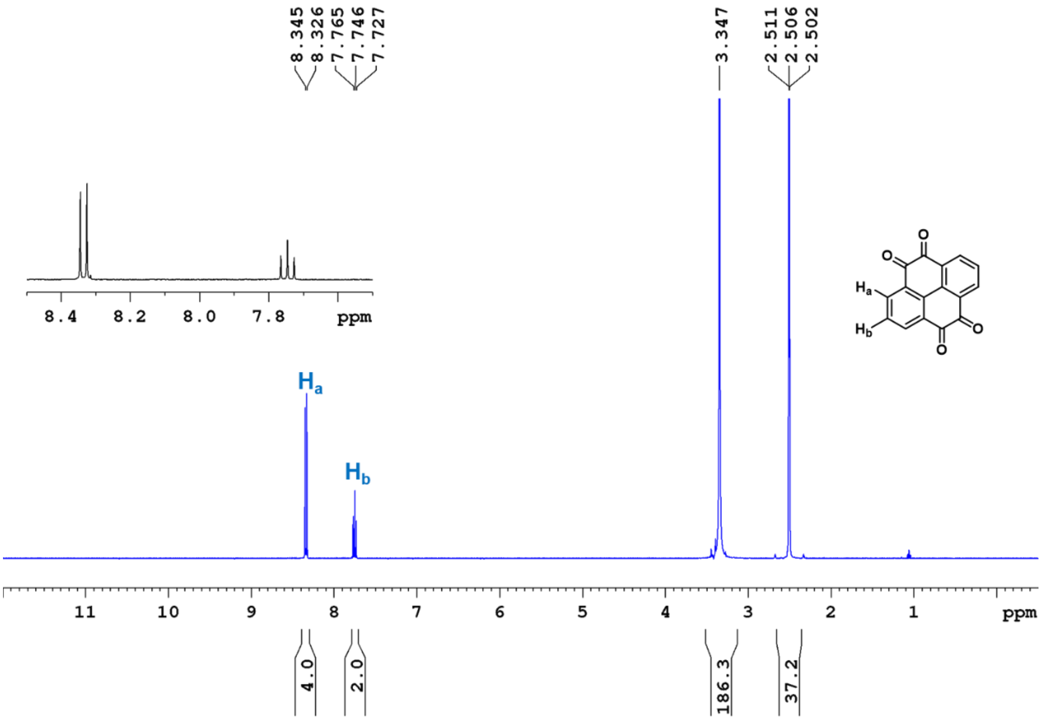

Figure S3. ^1H NMR (400 MHz, DMSO-d_6) spectrum of PT.

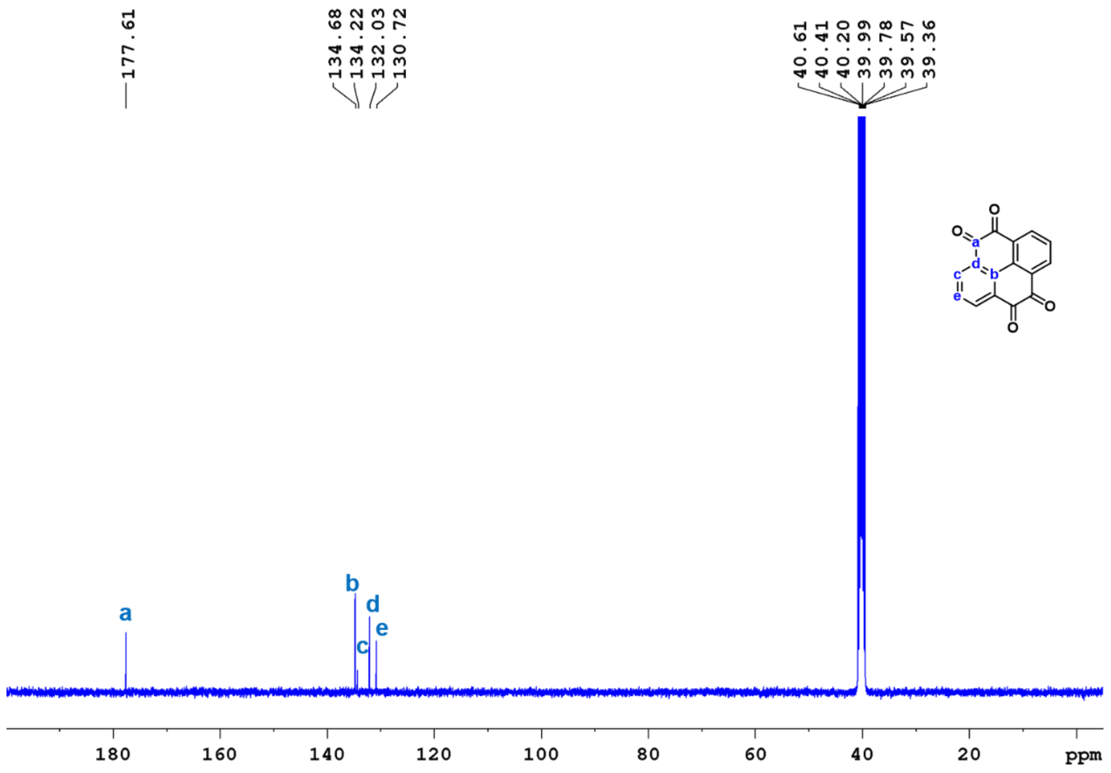

Figure S4. ^{13}C NMR (100 MHz, DMSO-d_6) spectrum of PT.

3.2 Synthesis of 2,7-Dinitropyrene-4,5,9,10-tetraone (DNPT)

DNPT was synthesized according to the reported protocol with slight modification.[3] PT (2 mM, 524 mg) was added into a 20 mL mixture of sulfuric acid and fuming nitric acid (4:1, v/v), and the mixture was heated at 110 °C overnight. The reaction mixture was cooled down to room temperature and poured into 200 mL of ice water. The suspension was neutralization by adding saturated $NaHCO_3$ solution. The yellow precipitate was then collected by filtering and washing with water several times. The solid was dried under vacuum at 65 °C for 24 h to afford 457 mg of DNPT as a yellow powder: 65% yield. ^{1}H NMR (400 MHz, DMSO-d_6, δ ppm): 8.89 (s, 4H). ^{13}C NMR (100 MHz, DMSO-d_6, δ ppm): 174.9, 149.2, 136.3, 134.4, 126.4. HRMS (EIS): calcd for ([$C_{16}H_4N_2O_8$]+Na)$^+$: m/z = 374.986; found: 374.9857.

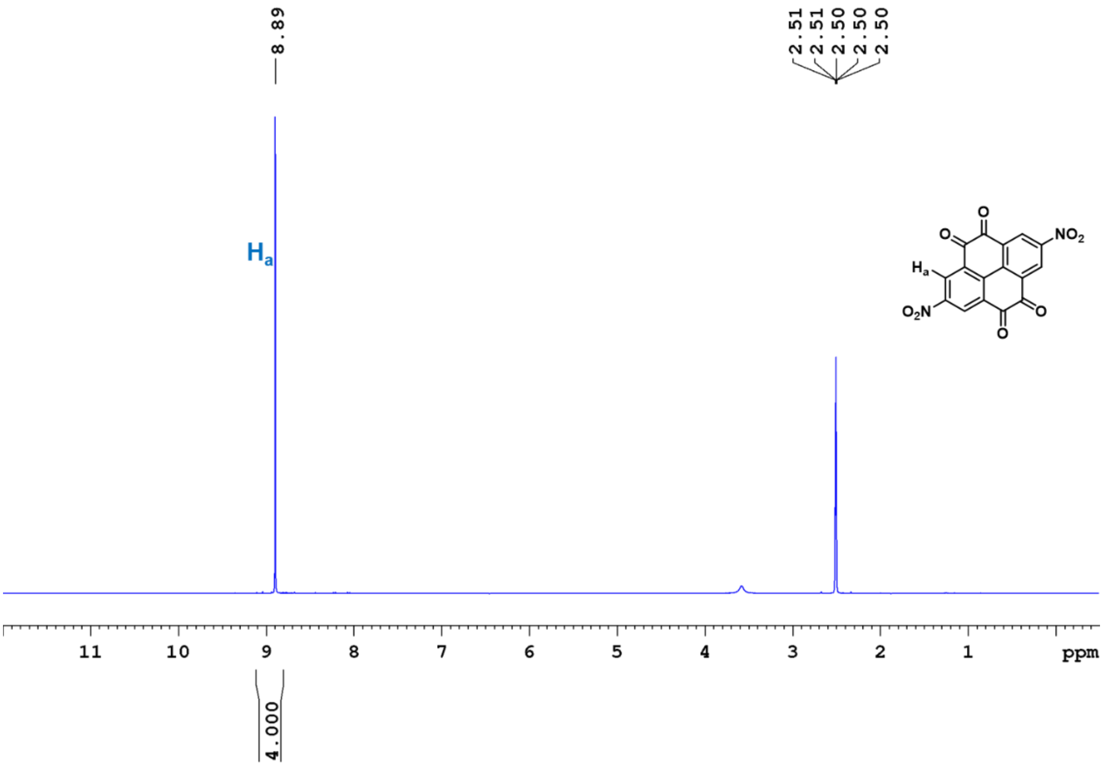

Figure S5. ^{1}H NMR (400 MHz, DMSO-d_6) spectrum of DNPT.

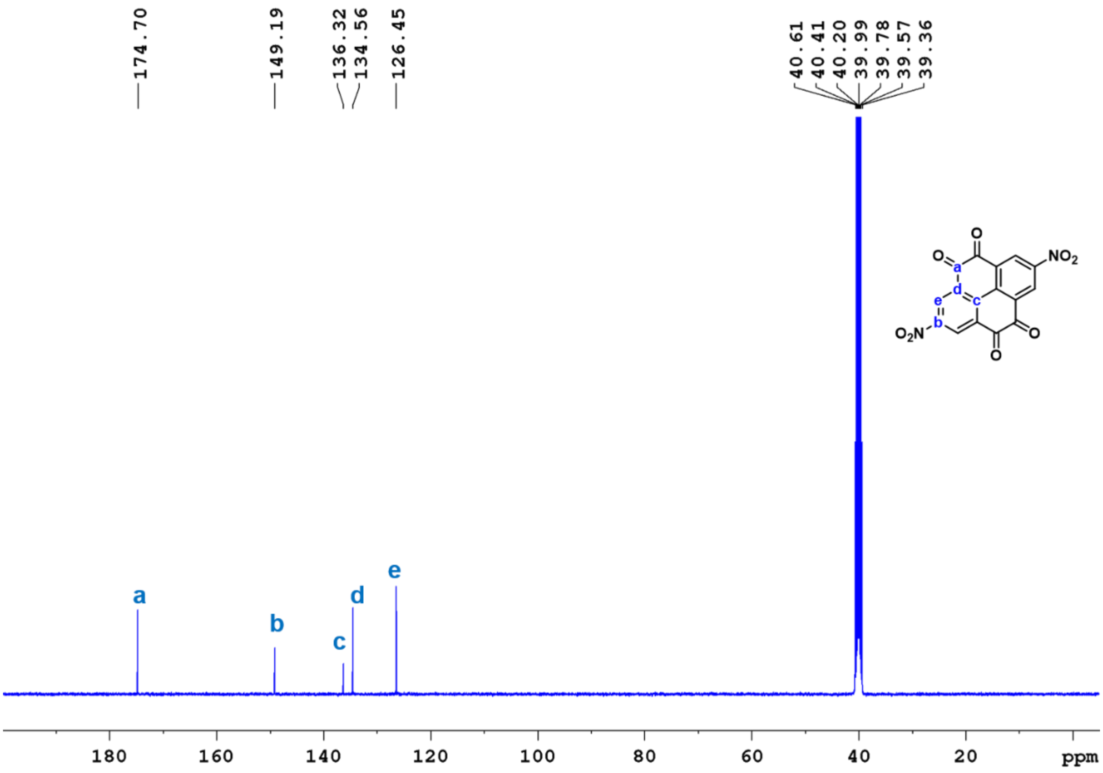

Figure S6. ^{13}C NMR (100 MHz, DMSO-d_6) spectrum of DNPT.

3.3 Synthesis of 2,7-Diaminopyrene-4,5,9,10-tetraone (DAPT)

DNPT (1 g, 2.8 mmol) was added in a 500mL two-necked flask. Sodium hydroxide (8.9 g, 224 mmol), water (150 mL), and sodium dithionite (4.4 g, 25.5 mmol) were added sequentially, and the reaction was heated to 50 °C. After stirring the reaction for 15 min at 50 °C, the reaction mixture was poured into saturated ammonium chloride solution (500 mL). The suspension was filtered, and the filtrate was washed with water to obtain a black powder. The black powder was dried under a vacuum at room temperature overnight to obtain the crude product. The crude product (457 mg), dichlorodicyanobenzoquinone (1.5 g), and methanol (30 mL) were stirred at 35 °C for 15 hours. Afterward, the reaction solution was diluted by adding ethyl acetate (60 mL). The suspension was removed by filtration and washed with ethyl acetate to afford 442 mg of DAPT as a black solid: 54% yield. ^{1}H NMR (400 MHz, DMSO-d_6, δ ppm): 7.37 (s, 4H), 5.96 (s, 4H). ^{13}C NMR (100 MHz, DMSO-d_6, δ ppm): 178.8, 149.1, 131.2, 124.9, 119.3.

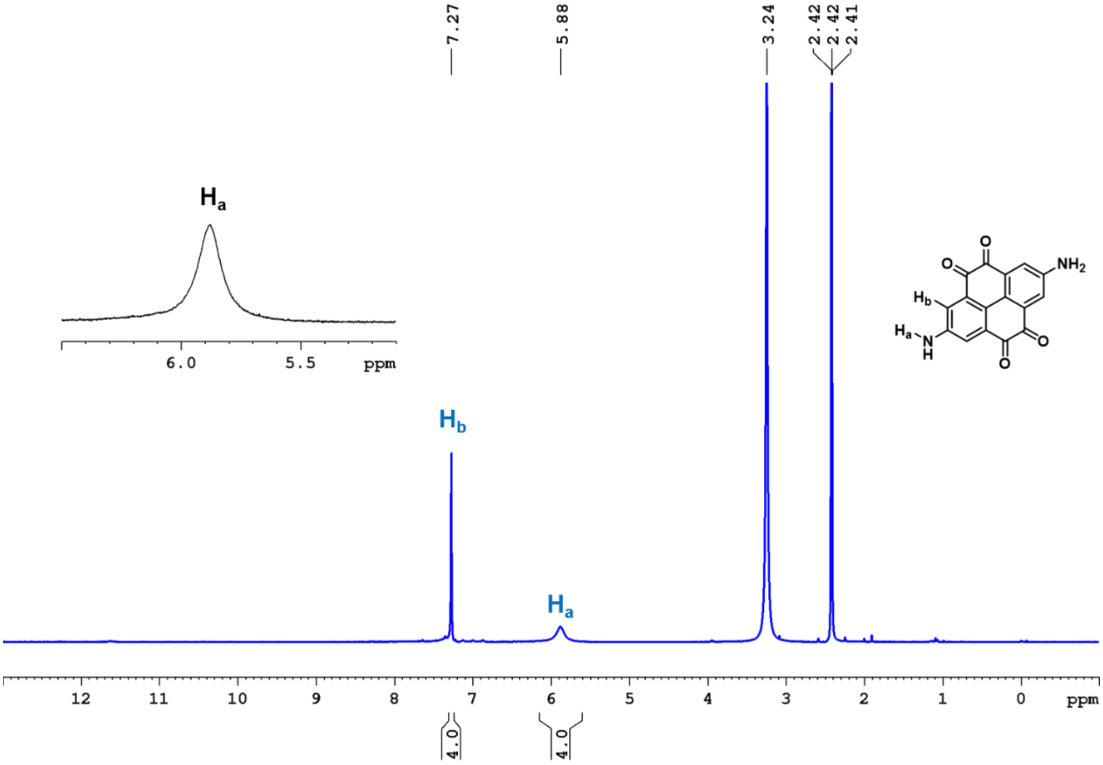

Figure S7. ^{1}H NMR (400 MHz, DMSO-d_6) spectrum of DAPT.

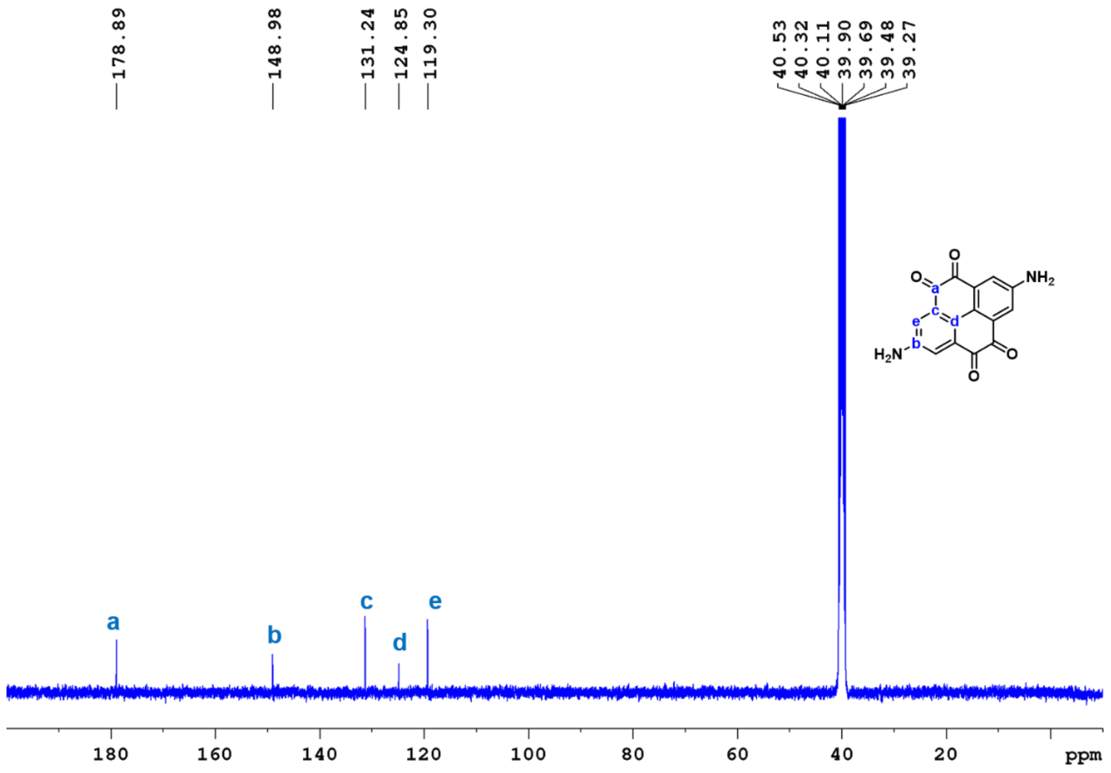

Figure S8. ^{13}C NMR (100 MHz, DMSO-d_6) spectrum of DAPT.

3.4 Synthesis of PT-COF

1,3,5-triformylphloroglucinol (TFG) (10.5 mg, 0.05 mmol), 2,7-diaminopyrene-4,5,9,10-tetraone (DAPT) (21.9 mg, 0.075 mmol), mesitylene (0.2 mL), 1,4-dioxane (0.8 mL), and aqueous acetic acid (0.2 mL, 6 M) were added to a 10 mL Pyrex tube (outer × inner diameter = 1.3 × 1.0 cm with a length 15 cm). This reaction mixture was homogenized by sonication for 15 minutes, and the Pyrex glass tube was subjected to three freeze-pump-thaw cycles and evacuated to an internal pressure of 100 mTorr. The tube was sealed off and then placed in an oven at 120 °C for 3 days. The black precipitate was collected by filtration and washed with DMF, DMSO, and acetone. The resulting solid was dried and then subjected to Soxhlet extraction with methanol as the solvent for 24 hours to remove the trapped guest molecules. The powder was collected and dried under reduced pressure at 85 °C to afford 28.2 mg of PT-COF as a black powder: 95% yield.

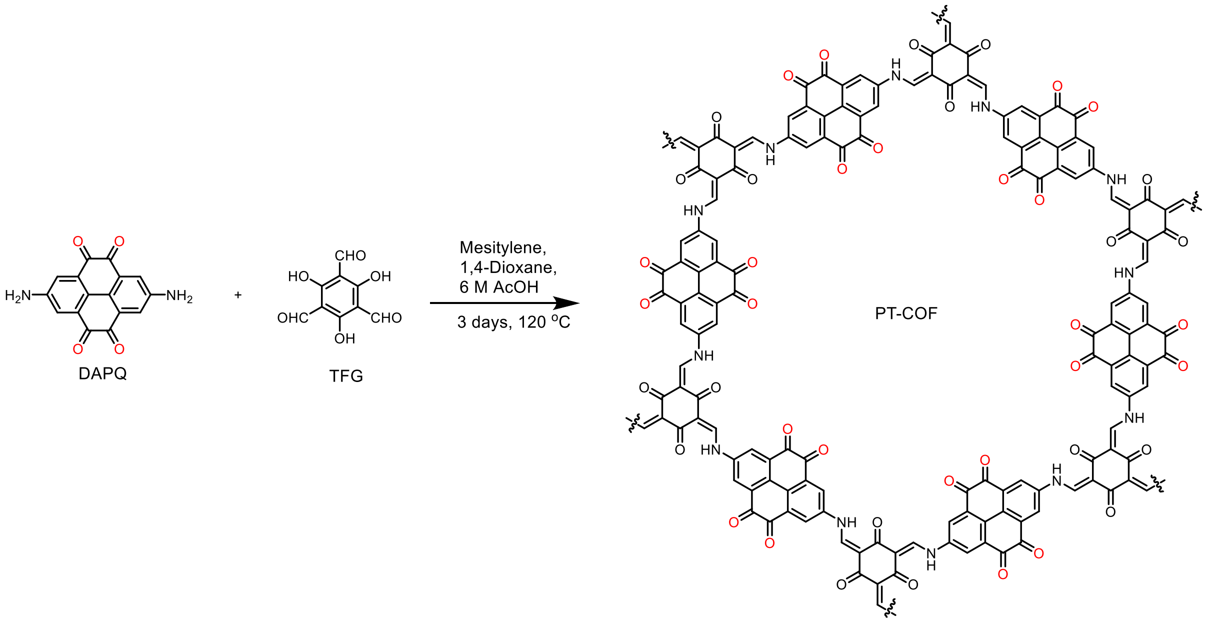

Scheme S2. Reaction scheme for the synthesis of PT-COF.

3.5 Synthesis of the PT-COFX Composites

A 10 mL Pyrex tube (outer × inner diameter = 1.3 × 1.0 cm with a length 15 cm) was charged with TFG (10.5 mg, 0.05 mmol), DAPT (21.9 mg, 0.075 mmol), CNT (10, 30, 50 wt.% of the composite based on the yield of the PT-COF), mesitylene (0.9 mL), 1,4-dioxane (0.3 mL), and aqueous acetic acid (0.2 mL, 6 M). This reaction mixture was homogenized by sonication for 30 minutes, and the Pyrex glass tube was subjected to three freeze-pump-thaw cycles and evacuated to an internal pressure of 100 mTorr. The tube was sealed off and then placed in an oven at 120 °C for 3 days. The black precipitate was collected by filtration and washed with DMF (3 × 5 mL), DMSO (3 × 5 mL), and acetone (3 × 5 mL). The resulting solid was dried and then subjected to Soxhlet extraction with methanol as the solvent for 24 hours to remove the trapped guest molecules. The powder was collected and dried under reduced pressure at 85 °C to afford the PT-COFX composites as black powders.

3.6 Optimization of Experimental Conditions

We repeated the synthesis of PT-COF using different reaction conditions to determine if they affected the crystallinity of the product (Table S1). From these reactions, we determined that using mesitylene (0.2 mL), 1,4-dioxane (0.8 mL), and aqueous acetic acid (0.2 mL, 6 M) afforded the most crystalline COF product and used these reaction conditions to synthesize the PT-COFX composites.

Table S1. Synthesis of PT-COF under different conditions

Entry[a]	Solvent (ml)	Acetic acid	Reaction temperature (°C)	Yield (%)	Crystallinity
1	mesitylene / 1,4-dioxane (0.9: 0.3)	0.1 ml, 3M	120	91	Crystalline
2	1,4-dioxane (1.0)	0.2 ml, 6M	120	91	Crystalline
3	mesitylene / 1,4-dioxane (0.2:0.8)	0.2 ml, 6M	120	95	Crystalline

[a]Aldehyde (0.05 mmol), diamine (0.075 mmol), 3 days.

3.7 Simulation X-ray Diffraction Patterns for COF Structures

Structural models of PT-COF were generated using the Accelrys3 Materials Studio (version 5.5) program suite.[4] Initially, an AA stacked model was generated with hexagonal *P*6/*m* space group symmetry. The *a* and *b* unit cell parameters were based on the distance between the centre of the trisubstituted rings in a structural model. The *c*-axis was based on a stacking distance between the COF layers of 3.4 Å. The AA stacked model was then optimized using the Geometry Optimization routine, which includes energy minimization and cell parameter optimization using a Universal Force Field. An AB stacked model was generated using the same approach but with hexagonal $P6_3$ symmetry and a *c* unit cell axis of 6.8 Å. Simulated PXRD patterns were calculated using the Reflex Plus module. The predicted PXRD pattern of the AA stacked model more closely matched the experimental PXRD data of PT-COF (Figure S10). The experimental PXRD data was refined by Pawley refinement in TOPAS Academic (Figure 1b).[5]

Table S2. Atomic coordinates and unit cell parameters of AA model for **PT-COF**.

PT-COF AA model				
Space group: *P*6/*m*<br>*a* = *b* = 30.0680 Å, *c* = 3.5538 Å.				
Atom Name	*x*	*y*	*z*	Occupancy
C1	0.31589	0.70187	-0.50000	1
C2	0.27917	0.64872	-0.50000	1
O1	0.29945	0.73273	-0.50000	1
C3	0.22604	0.63299	-0.50000	1
N1	0.18779	0.58337	-0.50000	1
H1	0.21605	0.66082	-0.50000	1
H2	0.19982	0.55894	-0.50000	1
C4	0.10666	0.50935	-0.50000	1
C5	0.05244	0.48343	-0.50000	1
C6	0.02721	0.51261	-0.50000	1
C7	0.05642	0.56705	-0.50000	1
C8	0.11056	0.59144	-0.50000	1
C9	0.13622	0.56311	-0.50000	1
C10	0.02307	0.42925	-0.50000	1
C11	0.03092	0.59577	-0.50000	1
O2	0.04535	0.40352	-0.50000	1
O3	0.05669	0.64378	-0.50000	1
H3	0.12516	0.48860	-0.50000	1
H4	0.13143	0.63046	-0.50000	1

3.8 Preparation of Electrodes and Coin Cells

The positive electrodes were prepared using the following procedure: The active material and carbon black (Super C65, IMERYS, particle size less than 50 nm) were ground by pestle and mortar for 30 minutes. The solid was transferred into a small vial that was equipped with a stirrer bar. Poly (vinylidene fluoride) solution (5 wt.% PVDF in NMP solution) was added to the vial. The mass ratio of active materials, carbon black, and PVDF was 6:3:1 (PT-COF) or 7:2:1 (PT-COFX). Additional NMP was added to adjust the viscosity to form a slurry. After stirring the suspensions for 12 h, finely dispersed slurries were obtained, and these were then coated onto aluminium foil substrates (thickness × width × height = 0.005 × 16 × 25 cm) using a doctor blade at a fixed thickness. The substrates were then dried at room temperature for 6 h followed by drying under vacuum pressure at 80 °C for 12 hours to afford dry films. The films were then punched into discs with diameters of 10 mm. The discs were dried overnight under

vacuum at 80 °C and transferred directly into a glovebox kept under an Ar atmosphere with H_2O and O_2 concentrations ≤ 0.1 ppm. The resulting electrode loadings were in the range of 0.3-0.6 mg cm^{-2}.

Coin cells (CR2025) were assembled inside an Ar-filled glovebox. Li foil with a diameter of 12 mm and a thickness of 0.38 mm was used as the counter electrode, and glass microfiber (Whatman GF/F) was used as the separator. A mixture of 1,3-dioxolane and 1,2-dimethoxyethane (1:1 vol/vol) containing 1 M lithium bis(trifluoromethanesulfonyl)imide (LiTFSI) was used as the primary electrolyte formulation (Duoduo Chemicals). After preparation, the coin cells were taken out of the glovebox and tested.

3.9 *Operando* Raman Microscopy

The free-standing working electrode was prepared using PT-COF as the active material using a procedure described previously.[6–8] The PT-COF, conductive carbon (Super C65), PVDF binder and dibutyl phthalate (DBP, as a plasticizer) were dispersed in acetone. The mass ratio of the four components was 4:1:2:3, respectively. After mixing, the slurry was coated onto a clean glass tile using a doctor blade with a controlled thickness of 60 µm. Following evaporation of the acetone, electrode discs (6 mm) were punched from the film and subsequently washed with diethyl ether to extract and remove the DBP. The free-standing electrode films were then dried under vacuum at 80 °C and transferred directly into the Ar glovebox.

The *operando* spectroelectrochemical cell (ECC-Opto-Std, El-Cell) was prepared and sealed under the Ar glovebox atmosphere before being transferred to the spectrometer. A polished Li foil disc (9 mm, 0.38 mm thick) was used as the counter/reference electrode and a glass microfibre (Whatman GF/F) disc wetted with the electrolyte (described above) was used as the separator. The hermetically sealed cell features an optical window permitting access for the Raman laser to focus on the rear side of the free-standing PT-COF working electrode, *via* holes of an Al mesh current collector. The *operando* cell was initially cycled to 3.4 V *vs*. Li^+/Li and held for 20-30 mins to achieve a fully charged state for one full discharge/charge cycle. The cell was then cycled at a rate of *ca*. C/5 (54 mA g^{-1}) to 1.6 V *vs*. Li^+/Li and then recharged back to 3.5 V *vs*. Li^+/Li. The cell was held at the lower voltage limit for 30 mins to promote complete lithiation of the active material. *Operando* Raman spectra of the PT-COF electrode were collected continuously in parallel with electrochemical cycling, wherein 1 spectrum (comprising multiple accumulations) was collected every *ca*. 3 mins during the experiment.

4. Supplementary Data

Solid-state NMR and FT-IR Spectra of PT-COF and the PT-COFX Composites

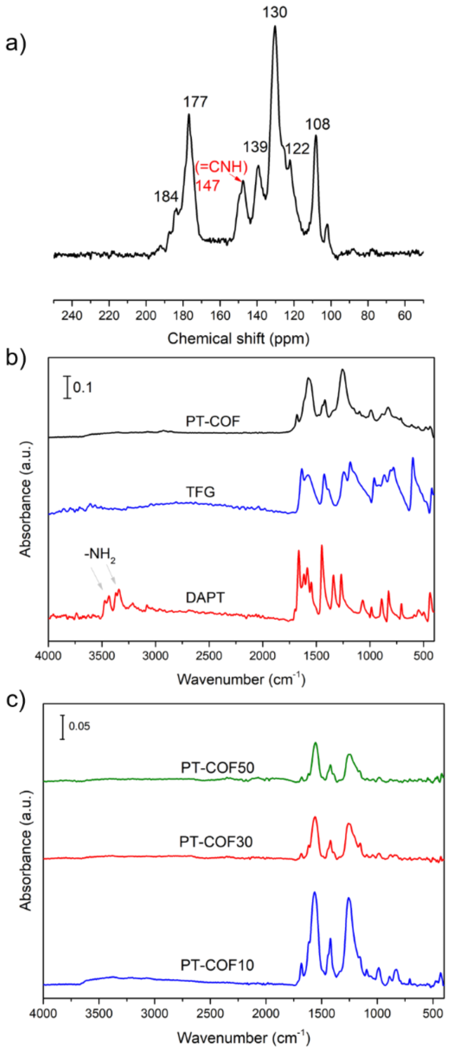

Figure S9. (a) ^{13}C solid-state NMR CP/MAS spectrum of PT-COF; (b) FT-IR spectra of TFG, DAPT, PT-COF; (c) FT-IR spectra of PT-COF10, PT-COF30, and PT-COF50. The peaks observed at 147 and 108 ppm in the PT-COF ^{13}C CP-MAS spectrum are assigned to the enamine carbon (=CNH) and α-enamine carbon, respectively, indicating the formation of β-ketoenamine-linked COF. FT-IR spectra of PT-COF and the PT-COFX composites show two characteristic peaks at 1259 and 1675 cm^{-1}, which were assigned to the stretching vibrations of β-ketoenamine C−N moieties and C=O of the DAPT unit, respectively. $\upsilon_{N\text{-}H}$ at 3470, 3437, 3367, and 3342 cm^{-1} in the FT-IR spectra of the DAPT precursor were not observed in the PT-COF, indicating the consumption of the DAPT in the reaction.

PXRD Patterns of PT-COF and the PT-COFX Composites

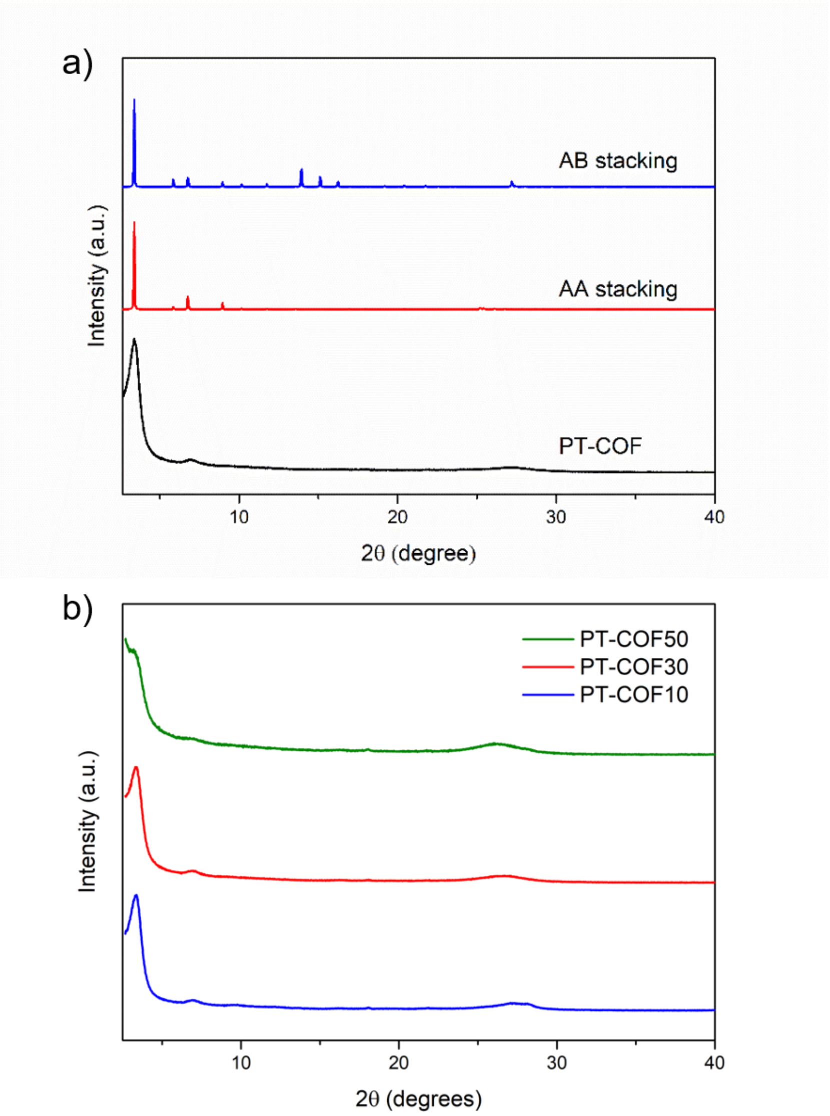

Figure S10. (a) Experimental PXRD pattern of PT-COF and the simulated PXRD patterns for AA and AB stacking models; (b) PXRD patterns of PT-COF10, PT-COF30, and PT-COF50. The more broad PXRD peak widths in the PT-COFX composites compared to the native PT-COF indicated the composites were less crystalline by PXRD alone, with PT-COF50 appearing the least crystalline. However, the TEM images (see Figures 1 and S13) show the expected trend of the COF layers in the core-shell composites becoming thinner as a greater wt.% of CNT was used to produce the PT-COFX composites, which is likely to contribute to the increased broadening of the PXRD peaks across the series.

Chemical Stability of PT-COF

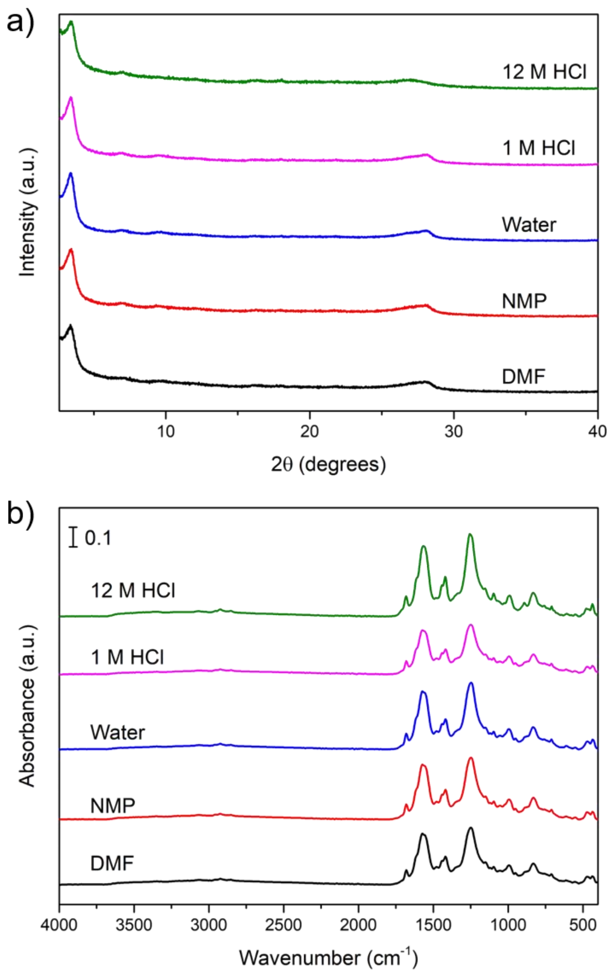

Figure S11. (a) PXRD and (b) FT-IR spectra of PT-COF after exposure to water, aqueous acid, *N*-dimethylformamide, and *N*-methyl-2-pyrrolidone for 48 hours.

Gas Sorption Isotherm and Pore Size Distribution

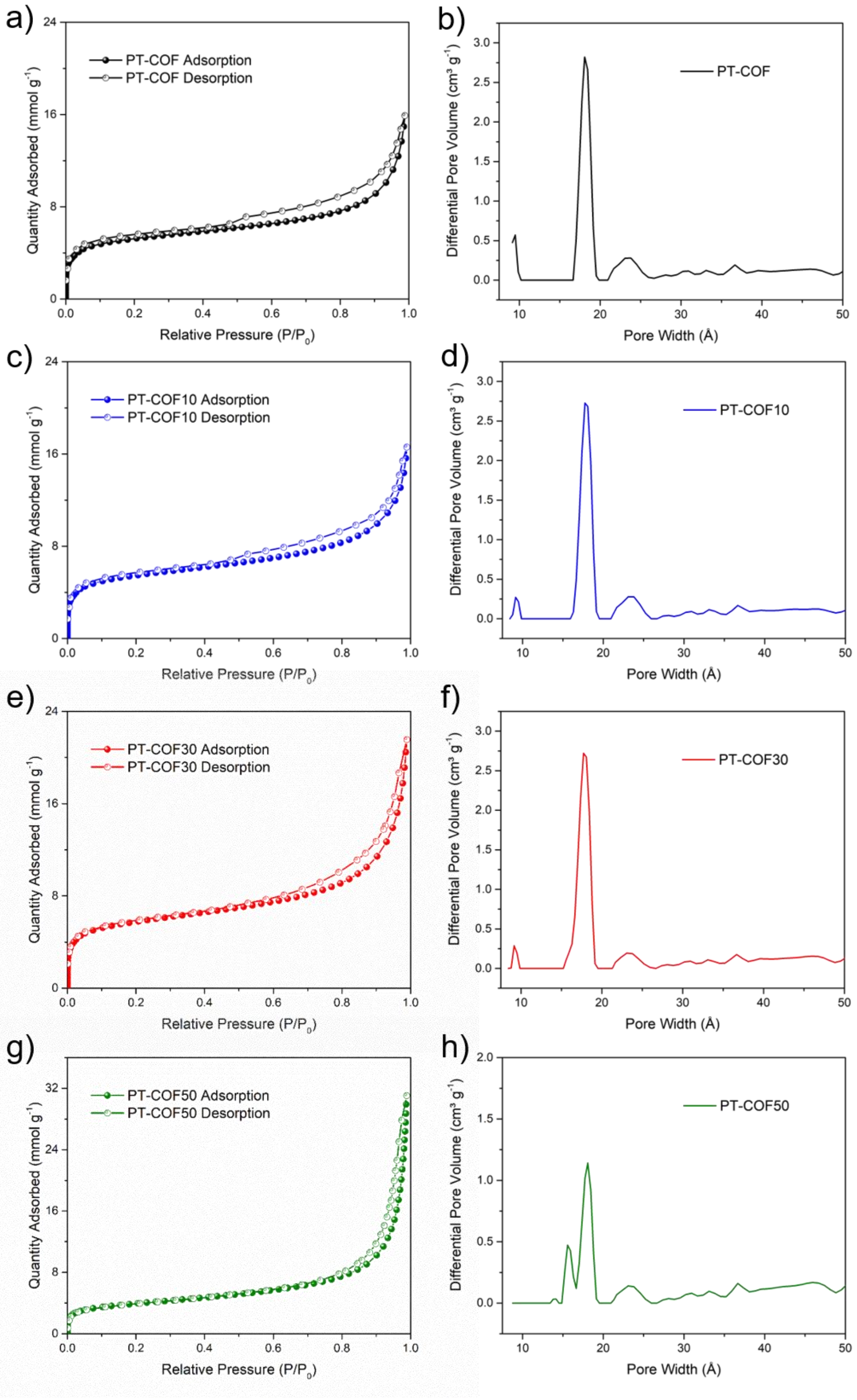

Figure S12. (a, c, e, g) N_2 sorption isotherms of PT-COF and the PT-COFX composites (77.3 K, solid symbols = adsorption; open symbols = desorption); (b, d, f, h) Pore size distribution plots of PT-COF and the PT-COFX composites (calculated by NL-DFT for pillared clay).

TEM Images of PT-COF10 and PT-COF30

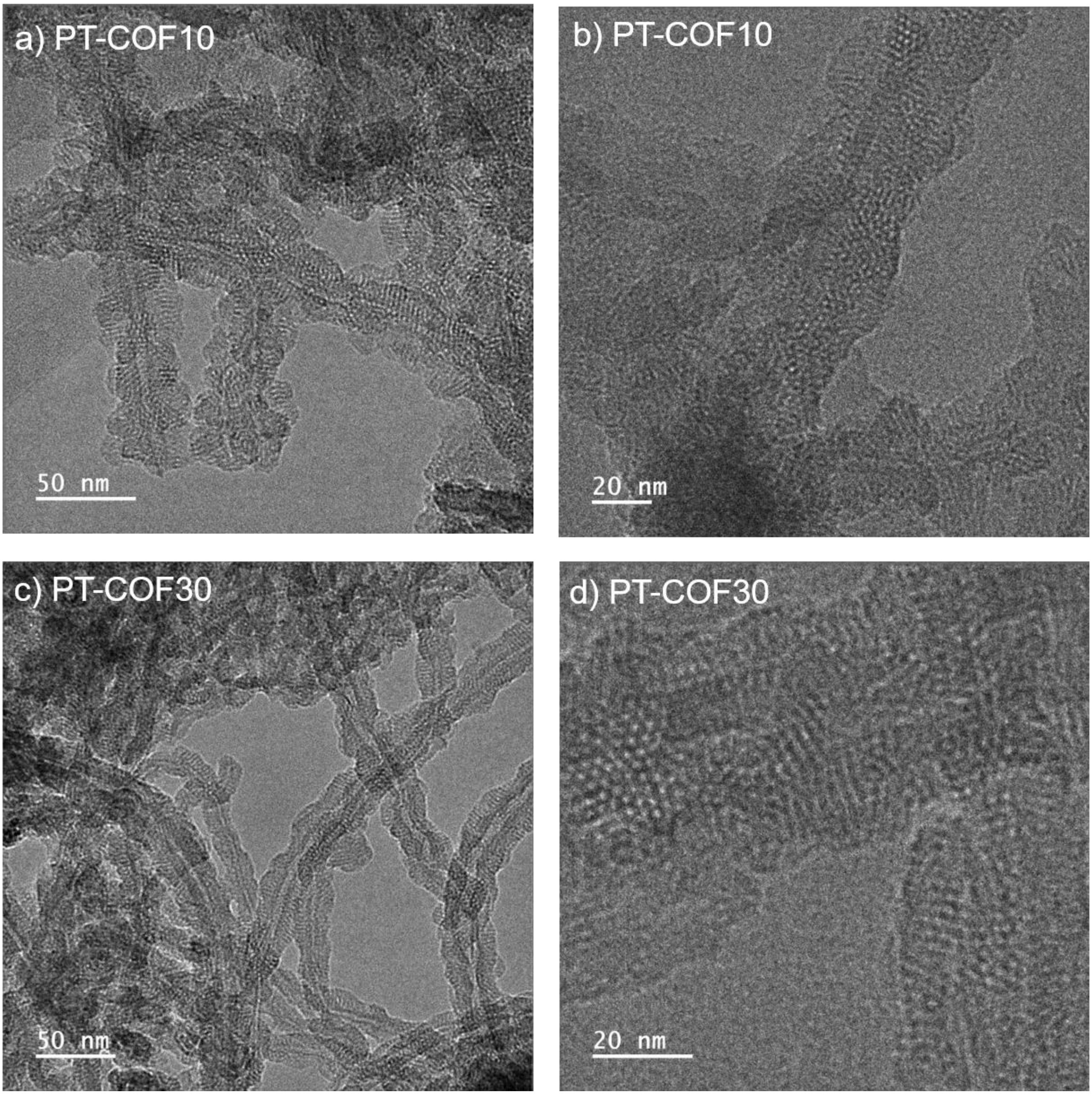

Figure S13. TEM images of PT-COF10 (a,b) and PT-COF30 (c,d). Scale bars are included.

Cycling Performance of PT-COF

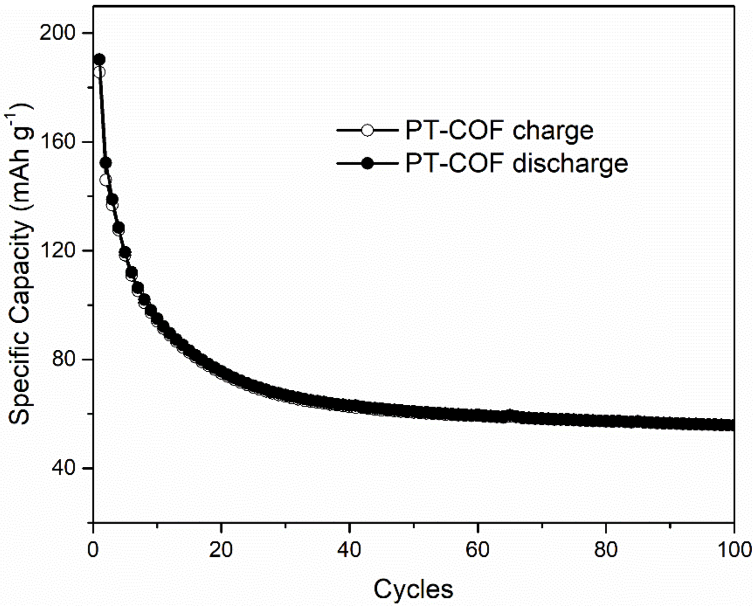

Figure S14. Cycling performance of PT-COF in a conventional Li-ion battery electrolyte (1 M $LiPF_6$ in EC/DMC (1:1 v/v) over 100 cycles at 100 mA g^{-1} operated in the voltage range of 1.5-3.5 V. The PT-COF based positive electrode in a carbonate-based electrolyte exhibits an initial capacity of 190 mA h g^{-1} but rapidly fades to 60 mAh g^{-1} within 50 cycles at the current density of 100 mA g^{-1}.

Capacity Contribution of CNT in the PT-COFX Composites

The specific capacities of the PT-COFX composites were calculated based on the mass of the PT-COF in the composite. However, both PT-COF and CNT contribute to the overall capacity of the electrode, so the total specific capacity of the PT-COFX composites was calculated using Equation S2:

$$C_{\mathrm{PT-COFX}} = \frac{a \times m \times C_{\mathrm{PT-COF}} + \mathrm{b} \times m \times C_{\mathrm{CNT}}}{a \times m} \qquad \textbf{Equation S2}$$

Where $C_{\mathbf{PT\text{-}COFX}}$ and $C_{\mathbf{PT\text{-}COF}}$ are the specific capacities of the PT-COFX composites and PT-COF, respectively, in the composite electrodes. C_{CNT} is the specific capacity of pure CNT (13 mAh g^{-1}) without PT-COF, and *m* is the mass of the PT-COFX composites, *a* and *b* are the contents of PT-COF and CNT in the PT-COFX, such that a+b=1. Therefore, the capacity contribution of CNT in the PT-COFX composites is $C_{\mathrm{PT\text{-}COFX}}$-$C_{\mathrm{PT\text{-}COF}}$ = $(b \times C_{\mathrm{CNT}})/a$

Table S3. Capacity contribution of CNT in the PT-COFX composites.

Sample	Calculation of capacity contribution of CNT in the PT-COFX composites / mAh g^{-1}
**PT-COF10**	0.1×13/0.9 = 1
**PT-COF30**	0.3×13/0.7 = 6
**PT-COF50**	0.5×13/0.5 = 13

Cycling Performance of the DAPT Monomer

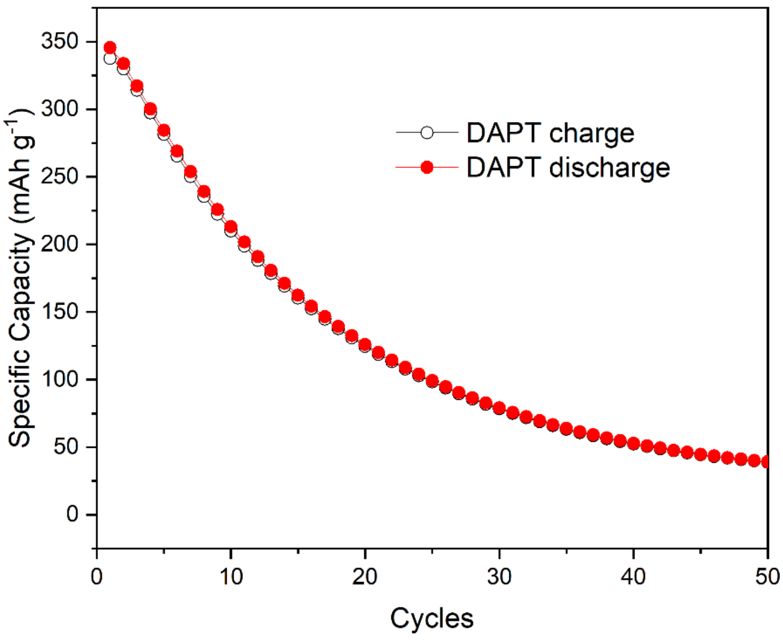

Figure S15. Cycling performance of the DAPT monomer over 50 cycles at 100 mA g^{-1} operated in the voltage range of 1.5-3.5 V. DAPT-based positive electrodes exhibit a capacity of 345 mAh g^{-1} at the current density of 100 mA g^{-1}, corresponding to 94% of its theoretical specific capacity of 367 mAh g^{-1}. The capacity is higher than that of the PT-COF and the PT-COFX composite electrodes. However, the capacity of DAPT monomer drops rapidly to 40 mAh g^{-1} in 50 cycles because DAPT is soluble in the organic electrolyte.

Table S4. Average discharge potential and cycling performance of the PT-COFX composites and other COFs-based composite cathodes reported previously in the literature.

Active compound	Key functional group	Q^a (mAh g^{-1}) / Current density (mA g^{-1})	Average discharge Voltage (*vs.* Li^+/Li /V)	Cycling performance	Rate capacity (mAh g^{-1}) / Current density (mA g^{-1})	Ref.
PT-COF50[b]	Carbonyl group	280 / 200	2.55	82% after 3000 cycles (2000 mA g^{-1})	229 / 5000	**This work**
PT-COF30	Carbonyl group	267 / 200	2.55	99% after 150 cycles (200 mA g^{-1})	145 / 5000	**This work**
PT-COF10	Carbonyl group	225 / 200	2.55	99% after 150 cycles (200 mA g^{-1})	115 / 5000	**This work**
PT-COF	Carbonyl group	193 / 200	2.55	99% after 150 cycles (200 mA g^{-1})	76 / 5000	**This work**
PIBN-G	Imide-benzoquinone	271 / 28	2.3	88% after 300 cycles (1400 mA g^{-1})	198 / 2800	9
DABQ-ECOF	Benzoquinone	210 / 20	2.8	—/—	—	10
DAAQ-ECOF	Anthraquinone	145 / 20	2.3	92% after 1800 cycles (500 mA g^{-1})	—	10
TEMPO-ECOF	Nitroxide radical	115 / 20	3.15	—/—	—	10
PPTODB	Pyrene-4,5,9,10-tetraone	198 / 20	2.5	68% after 150 cycles (20 mA g^{-1})	143 / 1000	11
PI-ECOF-1/rGO50	Imide	167 / 14.2	2.02	70% after 300 cycles (142 mA g^{-1})	100 / 1420	12
PI-ECOF-1	Imide	112 / 14.2	2.02	77% after 300 cycles (142 mA g^{-1})	0.2 / 1420	12
DAPQ-COF50	Phenanthrene-quinone	161 / 500	2.56	76% after 3000 cycles (2000 mA g^{-1})	93 / 50000	13
DAPQ-COF30	Phenanthrene-quinone	116 / 500	2.56	100% after 400 cycles (500 mA g^{-1})	104 / 2000	13
DAPQ-COF10	Phenanthrene-quinone	92 / 500	2.56	100% after 400 cycles (500 mA g^{-1})	70 / 2000	13
DAPQ-COF	Phenanthrene-quinone	73 / 500	2.56	100% after 400 cycles (500 mA g^{-1})	47 / 2000	13
AZO-1	Azo	140 / 73	~ 1.5	100% after 4500 cycles (1450 mA g^{-1})	63 / 5810	14

Table S4 cont. on page S24

AZO-1@CNT	Azo	157 / 73	~ 1.5	100% after 5000 cycles (5810 mA g^{-1})	92 / 5810	14
Tb-DANT-COF	Naphthimide	144/ 50	2.5	75% after 300 cycles (500 mA g^{-1})	67 / 2000	15
Tp-DANT-COF	Naphthimide	93/ 200	2.4	85% after 600 cycles (1000 mA g^{-1})	66 / 2000	15
2D CCP-HATN@CNT	Hexaaza-trinaphthalene	116 / 11.7	2.02	91% after 1000 cycles (500 mA g^{-1})	94 / 1000	16
E-TP-COF	C=O and C=N groups	110 / 200	-	87.3% after 500 cycles (200 mA g^{-1})	30 / 2000	17
2D-PAI@CNT	Naphthimide	104 / 100	2.42	100% after 8000 cycles (500 mA g^{-1})	96 / 2000	18
2D-PAI	Naphthimide	28 / 100	2.42	100% after 8000 cycles (500 mA g^{-1})	—	18
PEDOT@DAPH-TFP	Phenazine	94 / 85.5	2.3	31% after 500 cycles (171 mA g^{-1})	47 / 3420	19
DAPH-TFP	Phenazine	82 / 85.5	2.3	43% after 500 cycles (171 mA g^{-1})	49 / 3420	19
PEDOT@DAAQ-TFP	Anthraquinone	52 / 78.5	2.2	28% after 500 cycles (157 mA g^{-1})	32 / 3140	19
DAAQ-TFP	Anthraquinone	50 / 78.5	2.2	19% after 500 cycles (157 mA g^{-1})	17 / 3140	19
DTP-ANDI-COF@CNTs	Naphthimide	74 / 200	2.4	100% after 700 cycles (200 mA g^{-1})	63 / 1000	20

[a] The highest delivered reversible discharge capacity (Q).

[b] PT-COF50 shows nearly double the capacity compared to recently reported phenanthrene-quinone functionalized COF and CNT composite (DAPQ-COF50).[13] The capacity of PT-COF50 is comparable to the poly(imide-benzoquinone) COF and graphene composite (PINB-G) that exhibited a capacity of 271 mAh g^{-1} at 0.1 C (28 mA g^{-1}).[9] Additionally, PT-COF50 delivered a higher capacity than the thiazole-linked COF and CNT composite (AZO-1@CNT) that exhibited a discharge capacity of 157 mAh g^{-1} at 0.5 C,[14] and the 2D polyarylimide COF and CNT composite (2D-PAI@CNT) that exhibited a capacity of 104 mAh g^{-1} at 100 mA g^{-1}.

Electrochemical Performance of CNT and Carbon Black

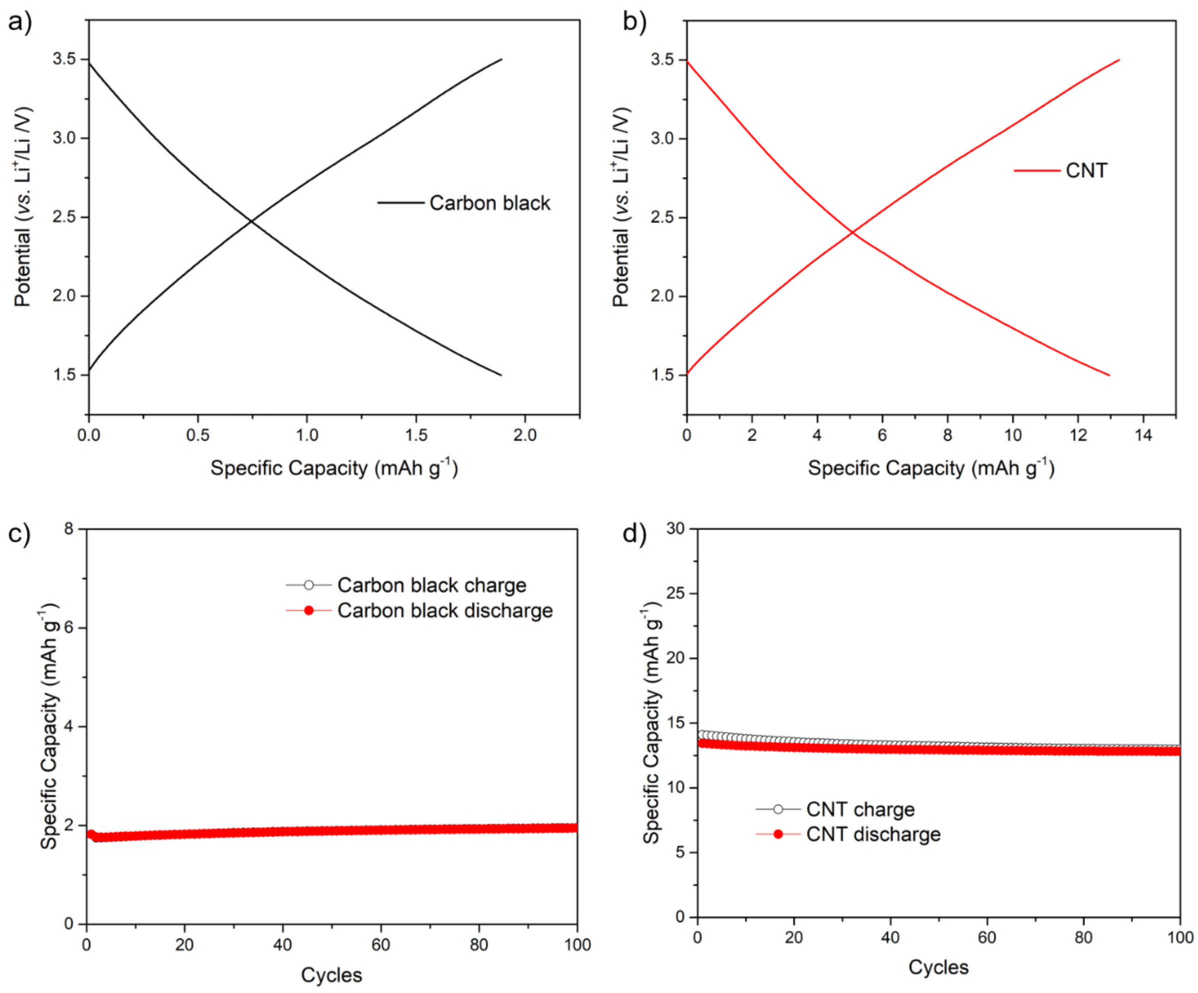

Figure S16. (a) Galvanostatic charge/discharge curve of carbon black electrode at 200 mA g^{-1}; (b) Galvanostatic charge/discharge curve of CNT electrode at 200 mA g^{-1}; (c) Cycling performance over 100 cycles for carbon black electrode at 200 mA g^{-1} operated in the voltage range of 1.5-3.5 V; (d) Cycling performance over 100 cycles for CNT electrode at 200 mA g^{-1} operated in the voltage range of 1.5-3.5V.

Charge-discharge Profiles of PT-COF and the PT-COFX Composites

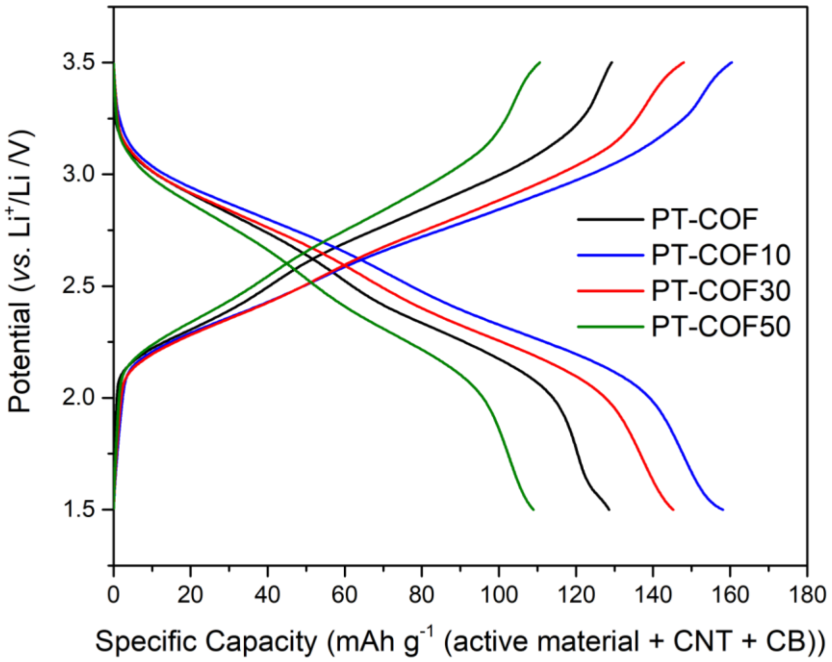

Figure S17. Charge-discharge profiles of PT-COF and the PT-COFX composites at 200 mA g^{-1} were calculated based on the sum mass of active materials, CNT and carbon black.

Electrochemical Impedance Characteristics of PT-COF and the PT-COFX Composites

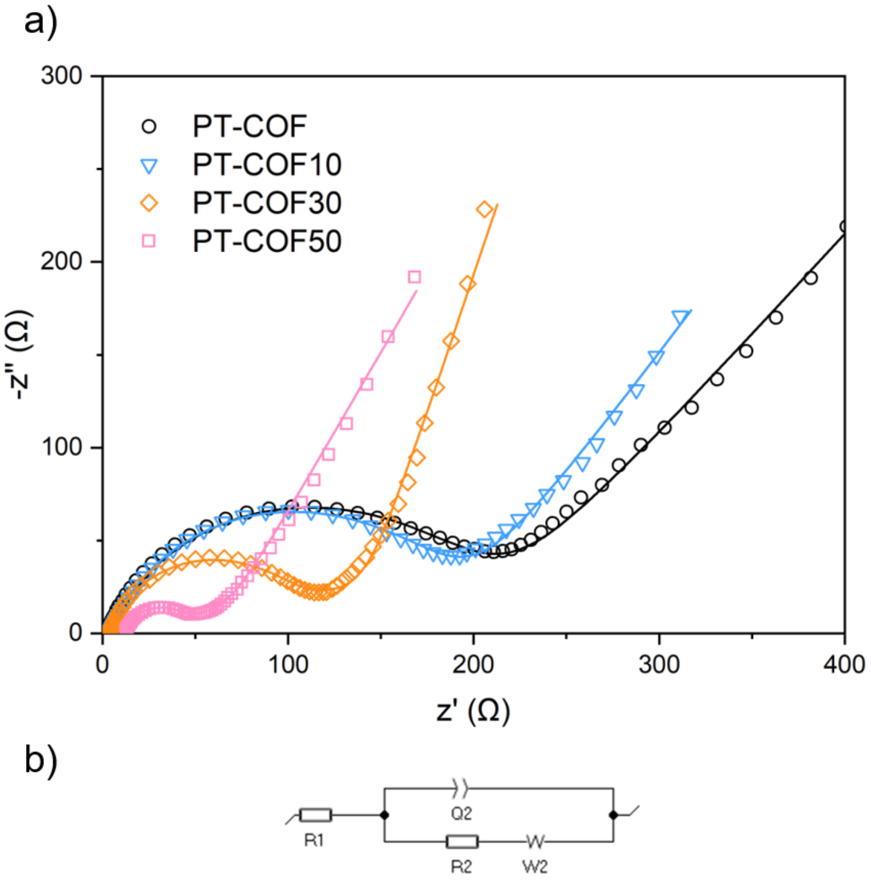

Figure S18. (a) Nyquist plots forth EIS measurements of the PT-COFX composite electrodes in half-cells *vs*. Li-metal. The impedance measurements were conducted at open circuit potential, before any electrochemical cycling, using a voltage perturbation of 10 mV within a frequency range of 100 mHz to 1 MHz. The solid lines represent the simulated data fitted using the equivalent circuit diagram below in EC-Lab (Biologic Science Instruments). (b) The equivalent circuit was used for fitting the electrochemical impedance data. The R_s (R1), R_{ct} (R2), CPE (Q2), and W (W2) represent ohmic resistance, charge-transfer resistance, constant phase element, and modified Warburg impedance, respectively.

Therein, the modified Warburg element is defined by the Equation S3 below, accounting for the restricted diffusion;

$$Z_{M_a}(f) = R_d \frac{\coth(\tau_d j 2\pi f)^{\alpha/2}}{(\tau_d j 2\pi f)^{\alpha/2}} \qquad \textbf{Equation S3}$$

wherein f is the frequency in Hz, $\alpha \in [0, 1]$ and j is the imaginary number $j^2 = -1$. τd is the diffusion time constant in s, and Rd is the resistance associated with the restricted linear diffusion mechanism.

The Nyquist plots, showing the trends in impedance characteristics for the composite materials as the proportion of CNTs utilized in the in situ polycondensation is increased, are provided in Figure S18. For all of the studied electrodes, the Nyquist plots consist of one depressed semicircle in the high-to-mid frequency range and a sloping line at mid-to-low frequencies. The semicircle reflects the charge-transfer resistance, R_{ct}, which is related to reaction kinetics, and the sloping line reflects the diffusion process of Li^+ ions in the electrode (Warburg impedance, W). The equivalent circuit used to simulate the impedance spectra is shown in Figure S18, and the obtained fitting parameters are shown in Table S5. Therein, in agreement with the reducing magnitude of the observed semicircle as the proportion of CNT in the composite material increases, the PT-COF50 material has the smallest R_{ct} (27.2 Ω) of the measured PT-COF and the PT-COFX composite electrodes. While the total proportion of CNTs in the electrode will affect bulk conductivities, these observed trends in R_{ct} support the assertion of improved charge transfer in PT-COF50 due to optimization of contact between the PT-COF and conductive carbon network.

Table S5. Fitted values of elements in the equivalent circuit for EIS data of PT-COF and the PT-COFX composite electrodes.

	**R_s (Ω)**	**CPE (F)**	**R_{ct} (Ω)**	**W(Ω)**
**PT-COF**	2.3	1.9×10^{-6}	189.3	55.1
**PT-COF10**	3.9	4.6×10^{-7}	163.9	84.2
**PT-COF30**	3.7	3.9×10^{-6}	100.1	96.1
**PT-COF50**	12.9	4.1×10^{-6}	27.2	71.8

FT-IR Characterization

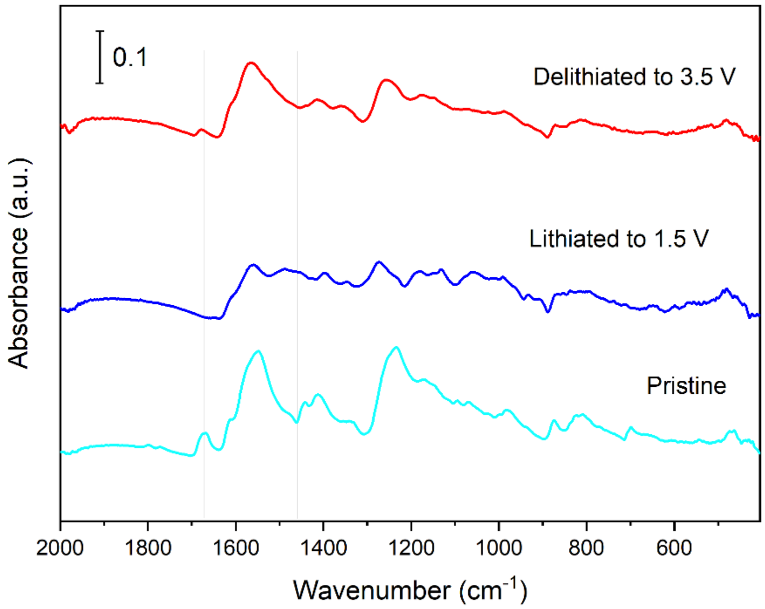

Figure S19. FT-IR spectra of PT-COF electrode materials at different states of lithiation/delithiation (pristine, lithiated to 1.5 V and delithiated to 3.5 V.

Electrochemistry of the DAPT Monomer

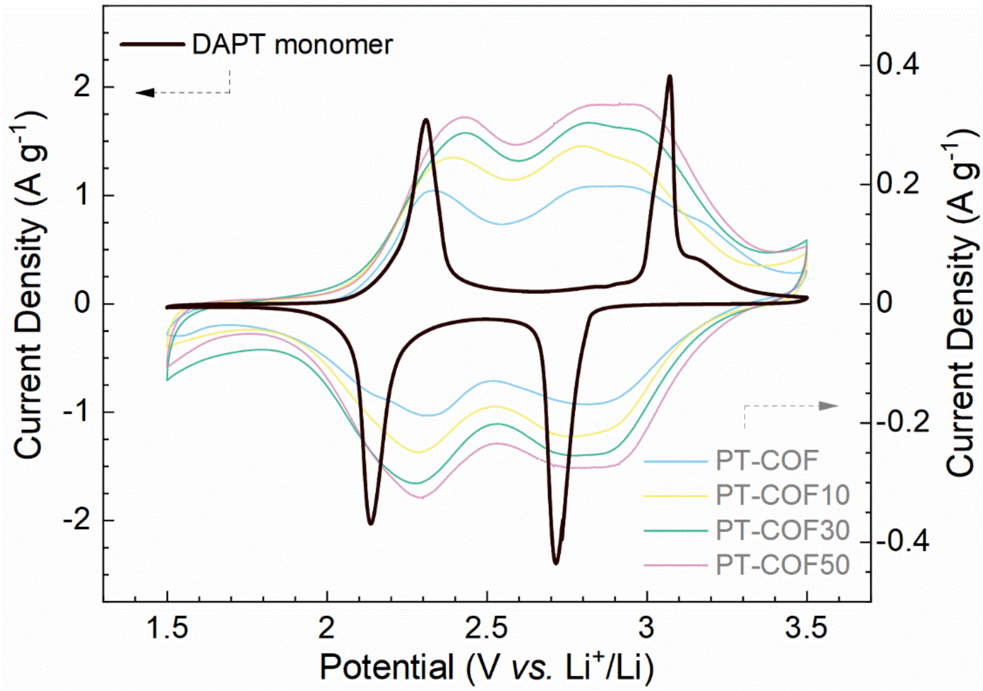

Figure S20. Cyclic voltammetry profiles of the PT-COFX composites and DAPT monomer based electrode at a scan rate of 0.5 mV s^{-1}.

Kinetic Study

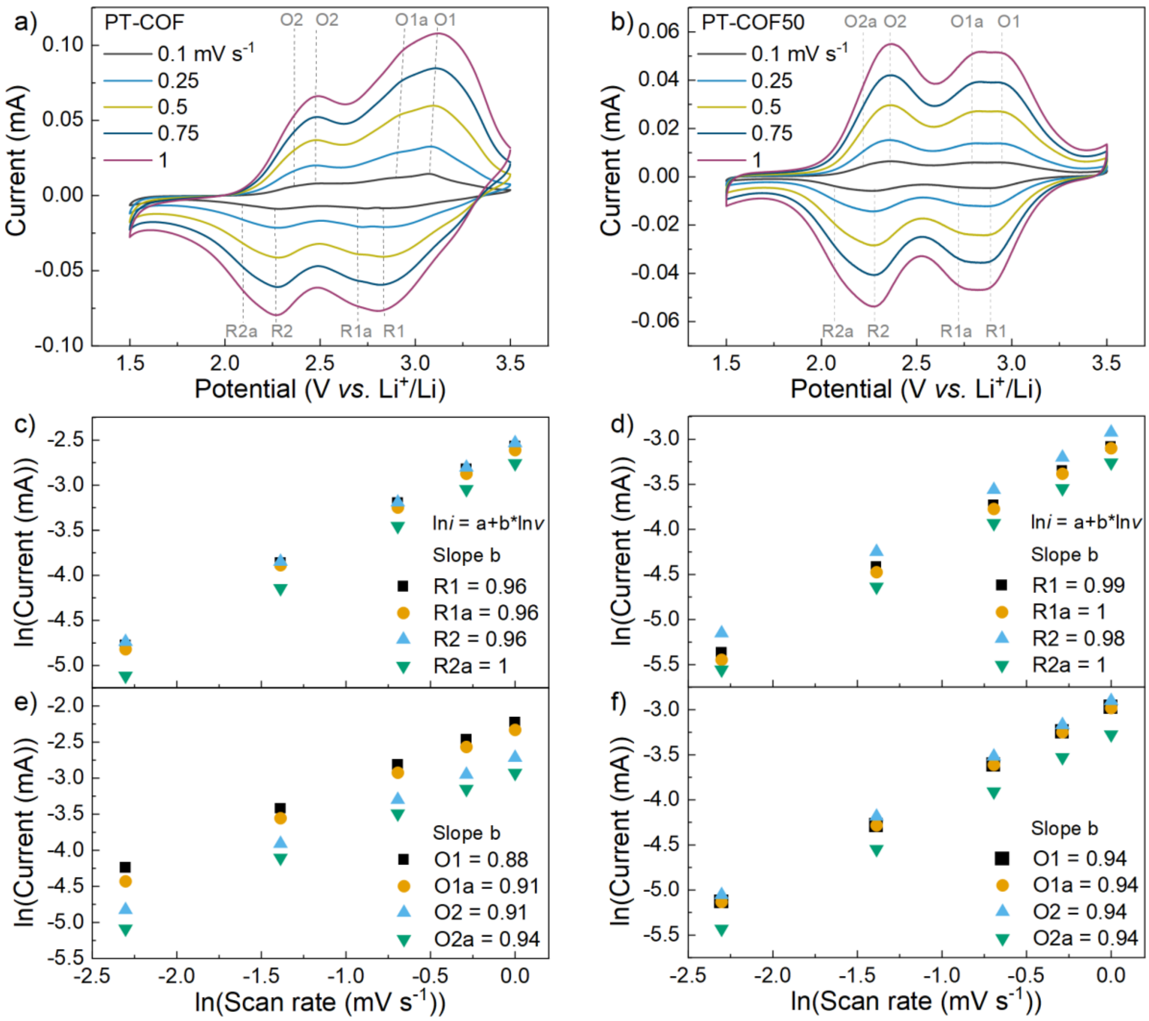

Figure S21. CVs of PT-COF (a) and PT-COF50 (b) at different sweep rates. (c-f) ln*i* *vs.* ln*v* plots (where *i* and *v* are the currents and the CV sweep rates, respectively) to determine the b values of reduction peaks (R1, R1a, R2, R2a) and oxidation peaks (O1, O1a, O2, O2a) for PT-COF (c, e) and PT-COF50 (d, f).

The electrochemical reaction kinetics of PT-COF50 were investigated by scanning the CV at different sweep rates (Figure S21). The current response (*i*) of PT-COF50 to the applied sweep rate (v, 0.1–1.0 mV s^{-1}) was recorded. According to the power law, $i = av^b$, b-values close to 0.5 indicate that the current is controlled by semi-infinite linear diffusion, by contrast, b-values close to 1 indicate that the current is surface-controlled. It is found that the b-values of eight redox peaks R1, R1a, R2, R2a, O1, O1a, O2, and O2a (0.99, 1.00, 0.98, 1.00, 0.94, 0.94, 0.94 and 0.94) are all close to 1 (Figure S21b and c), indicating that the charge storage in PT-COF50 is a fast surface-controlled process, which results from the high reactivity of carbonyl redox-active groups in PT-COF50 not limited by ion diffusion within the studied sweep range.

Fitted Raman Spectra

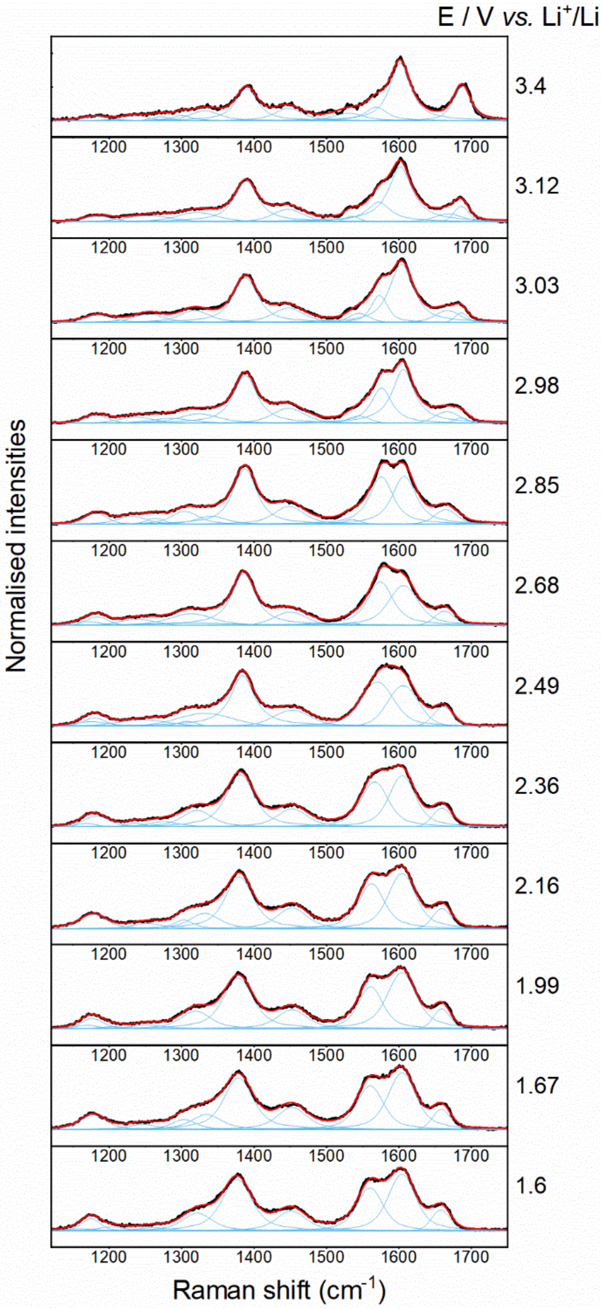

Figure S22. Fitted Raman spectra (baseline subtracted, normalized) used to extract peak shift information during the *operando* discharge of the PT-COF electrode at distinct cell voltages (values shown to the right of each panel). The black trace shows the experimental data while the blue and red traces show the simulated peaks and the cumulative fit, respectively.

Rationalizing the 4 Step Mechanism Proposal

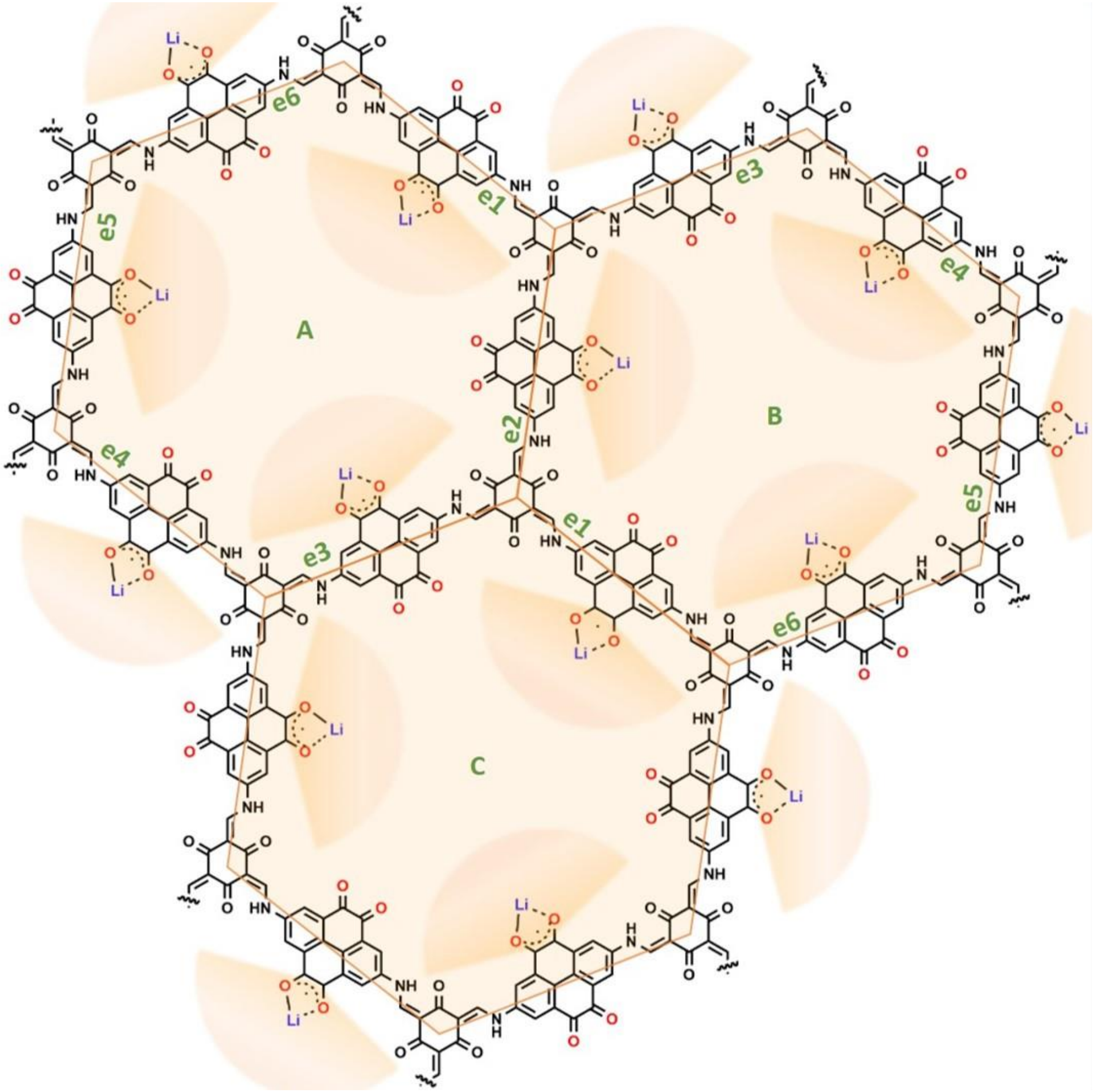

Figure S23. Schematic illustrating a small section of the PT-COF layer and an approximation of the distribution of shared delocalized lithium enolate intermediates following the first electron reduction step during the discharge in the Li^{+} containing electrolyte. The edges of hexagons A and B are labelled "e1", "e2" etc. for reference to discussion in the main text and shaded areas highlight the electrostatic repulsion these groups may incur on the adjacent unreacted 1,2-diketone groups.

The image (Figure S23) is provided to illustrate the likely distribution of reacted sites for the first electron reduction step clearly observed in the cyclic voltammetry of the PT-COFX composite materials (*i.e.*, e1, e3, and e5 in hexagon A, and e2, e4, and e6 in hexagon B). The rigid structure of the repeating active units within the PT-COF structure ensures that the charge distribution of adjacent reacted groups (*e.g.*, e1 and e3 in hexagon A), and the resulting electrostatic repulsion, increases the energy barrier to reducing the unreacted 1,2-diketone group at e2 within hexagon A. This barrier is not observed within the monomeric form of the DAPT unit (see Figure S20) since, in a randomly distributed collection of discrete DAPT molecules, both the 1,2-diketone units of the DAPT would not experience any constant steric or charge hindrance from surrounding DAPT molecules.

Effect of Electrolyte Wetting on the PT-COF

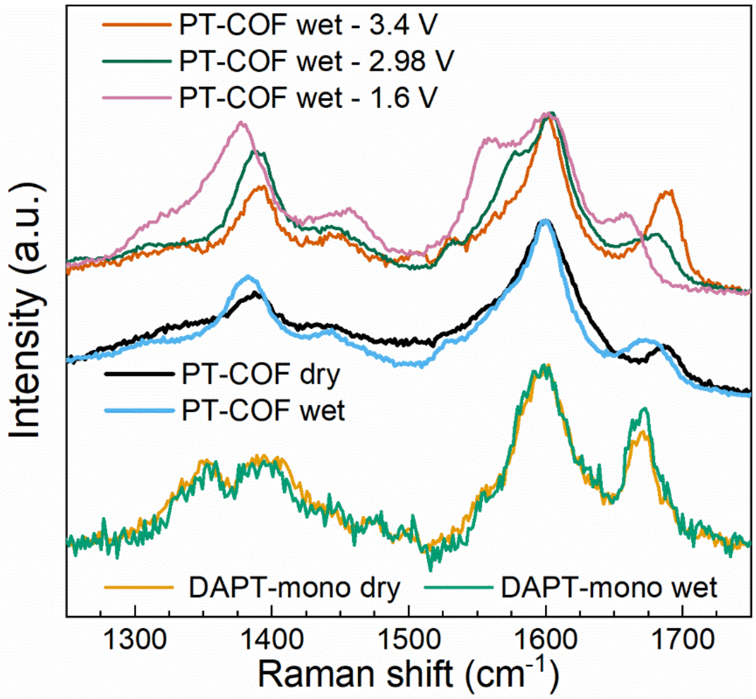

Figure S24. Raman spectra (baseline subtracted, normalized, and stacked by group) of electrodes based on the monomer (DAPT-mono, bottom pair) and the COF (PT-COF, middle pair) measured either with or without wetting of the electrolyte (*i.e.*, wet or dry, respectively). The top traces show a selection of *operando* collected Raman spectra of the PT-COF electrode at different voltages during the discharge.

As described in the main text, wetting of the PT-COF electrode with the electrolyte formulation results in a considerable shift of the C=O stretching mode compared to the dry, pristine material. Interestingly the same shift is not observed for the dry and wetted electrodes based on the DAPT monomer. The shift in the C=O stretching mode in the PT-COF material is comparable to that observed during the discharge process in the *operando* electrochemical cell. Critically however, unlike the discharging electrode (top spectra), this shift is not coupled with any changes in the C=C stretching mode around 1576-1560 cm^{-1} that arise from the electrochemical reduction process. Therein, it is suggested that the shift in the Raman mode arises from weak coordination with the partially desolvated Li^{+} cations as these infiltrate the pores/channels of the COF network.

5. References

(1) Letizia, J. A.; Cronin, S.; Ortiz, R. P.; Facchetti, A.; Ratner, M. A.; Marks, T. J. Phenacyl-Thiophene and Quinone Semiconductors Designed for Solution Processability and Air-Stability in High Mobility n-Channel Field-Effect Transistors. *Chem. A Eur. J.* **2010**, *16* (6), 1911–1928.

(2) Xing, Q.; Song, K.; Liang, T.; Liu, Q.; Sun, W. H.; Redshaw, C. Synthesis, Characterization and Ethylene Polymerization Behaviour of Binuclear Nickel Halides Bearing 4,5,9,10-Tetra(Arylimino)Pyrenylidenes. *Dalton Trans.* **2014**, *43* (21), 7830–7837.

(3) Li, Q.; Wang, H.; Wang, H. guo; Si, Z.; Li, C.; Bai, J. A Self-Polymerized Nitro-Substituted Conjugated Carbonyl Compound as High-Performance Cathode for Lithium-Organic Batteries. *ChemSusChem* **2020**, *13* (9), 2449–2456.

(4) Accelrys Materials Studio, version 5.5, Accelrys Software Inc.: San Diego, CA.

(5) Coelho, A. TOPAS-Academic. Coelho Software: Brisbane, Australia 2012.

(6) Cabo-Fernandez, L.; Mueller, F.; Passerini, S.; Hardwick, L. J. In Situ Raman Spectroscopy of Carbon-Coated ZnFe2O4 Anode Material in Li-Ion Batteries - Investigation of SEI Growth. *Chem. Commun.* **2016**, *52* (20), 3970–3973.

(7) Sole, C.; Drewett, N. E.; Hardwick, L. J. Insitu Raman Study of Lithium-Ion Intercalation into Microcrystalline Graphite. *Faraday Discuss.* **2014**, *172*, 223–237.

(8) Cabo-Fernandez, L.; Bresser, D.; Braga, F.; Passerini, S.; Hardwick, L. J. In-Situ Electrochemical SHINERS Investigation of SEI Composition on Carbon-Coated Zn0.9Fe0.1O Anode for Lithium-Ion Batteries. *Batter. Supercaps* **2019**, *2* (2), 168–177.

(9) Luo, Z.; Liu, L.; Ning, J.; Lei, K.; Lu, Y.; Li, F.; Chen, J. A Microporous Covalent-Organic Framework with Abundant Accessible Carbonyl Groups for Lithium-Ion Batteries. *Angew. Chemie Int. Ed.* **2018**, *57* (30), 9443–9446.

(10) Wang, S.; Wang, Q.; Shao, P.; Han, Y.; Gao, X.; Ma, L.; Yuan, S.; Ma, X.; Zhou, J.; Feng, X.; Wang, B. Exfoliation of Covalent Organic Frameworks into Few-Layer Redox-Active Nanosheets as Cathode Materials for Lithium-Ion Batteries. *J. Am. Chem. Soc.* **2017**, *139* (12), 4258–4261.

(11) Yao, C. J.; Wu, Z.; Xie, J.; Yu, F.; Guo, W.; Xu, Z. J.; Li, D. S.; Zhang, S.; Zhang, Q. Two-Dimensional (2D) Covalent Organic Framework as Efficient Cathode for Binder-Free Lithium-Ion Battery. *ChemSusChem* **2020**, *13* (9), 2457–2463.

(12) Wang, Z.; Li, Y.; Liu, P.; Qi, Q.; Zhang, F.; Lu, G.; Zhao, X.; Huang, X. Few Layer

Covalent Organic Frameworks with Graphene Sheets as Cathode Materials for Lithium-Ion Batteries. *Nanoscale* **2019**, *11* (12), 5330–5335.

(13) Gao, H.; Zhu, Q.; Neale, A. R.; Bahri, M.; Wang, X.; Yang, H.; Liu, L.; Clowes, R.; Browning, N. D.; Sprick, R. S.; Little, M. A.; Hardwick, L. J.; Cooper, A. I. Integrated Covalent Organic Framework/Carbon Nanotube Composite as Li-Ion Positive Electrode with Ultra-High Rate Performance. *Adv. Energy Mater.* **2021**, *11* (39), *2101880*.

(14) Singh, V.; Kim, J.; Kang, B.; Moon, J.; Kim, S.; Kim, W. Y.; Byon, H. R. Thiazole-Linked Covalent Organic Framework Promoting Fast Two-Electron Transfer for Lithium-Organic Batteries. *Adv. Energy Mater.* **2021**, *11* (17), 1–10.

(15) Yang, D. H.; Yao, Z. Q.; Wu, D.; Zhang, Y. H.; Zhou, Z.; Bu, X. H. Structure-Modulated Crystalline Covalent Organic Frameworks as High-Rate Cathodes for Li-Ion Batteries. *J. Mater. Chem. A* **2016**, *4* (47), 18621–18627.

(16) Xu, S.; Wang, G.; Biswal, B. P.; Addicoat, M.; Paasch, S.; Sheng, W.; Zhuang, X.; Brunner, E.; Heine, T.; Berger, R.; Feng, X. A Nitrogen-Rich 2D Sp 2 -Carbon-Linked Conjugated Polymer Framework as a High-Performance Cathode for Lithium-Ion Batteries. *Angew. Chem., Int. Ed.* **2019**, *58* (3), 849–853.

(17) Zhao, G.; Li, H.; Gao, Z.; Xu, L.; Mei, Z.; Cai, S.; Liu, T.; Yang, X.; Guo, H.; Sun, X. Dual-Active-Center of Polyimide and Triazine Modified Atomic-Layer Covalent Organic Frameworks for High-Performance Li Storage. *Adv. Funct. Mater.* **2021**, *31* (29), 2101019.

(18) Wang, G.; Chandrasekhar, N.; Biswal, B. P.; Becker, D.; Paasch, S.; Brunner, E.; Addicoat, M.; Yu, M.; Berger, R.; Feng, X. A Crystalline, 2D Polyarylimide Cathode for Ultrastable and Ultrafast Li Storage. *Adv. Mater.* **2019**, *31* (28), 1901478.

(19) Vitaku, E.; Gannett, C. N.; Carpenter, K. L.; Shen, L.; Abruña, H. D.; Dichtel, W. R. Phenazine-Based Covalent Organic Framework Cathode Materials with High Energy and Power Densities. *J. Am. Chem. Soc.* **2020**, *142* (1), 16–20.

(20) Xu, F.; Jin, S.; Zhong, H.; Wu, D.; Yang, X.; Chen, X.; Wei, H.; Fu, R.; Jiang, D. Electrochemically Active, Crystalline, Mesoporous Covalent Organic Frameworks on Carbon Nanotubes for Synergistic Lithium-Ion Battery Energy Storage. *Sci. Rep.* **2015**, *5* (1), 8225.
